# Machine Learning in Adapted Physical Activity: Clinical Applications, Monitoring, and Implementation Pathways for Personalized Exercise in Chronic Conditions: A Narrative Review

**DOI:** 10.3390/jfmk11010106

**Published:** 2026-03-04

**Authors:** Gianpiero Greco, Alessandro Petrelli, Luca Poli, Francesco Fischetti, Stefania Cataldi

**Affiliations:** 1 Department of Translational Biomedicine and Neuroscience (DiBraiN), University of Bari Aldo Moro, 70124 Bari, Italy; gianpiero.greco@uniba.it (G.G.); francesco.fischetti@uniba.it (F.F.); 2 Department of Neurosciences, Biomedicine and Movement Sciences, University of Verona, 37129 Verona, Italy; 3 Department of Education and Sport Sciences, Pegaso Telematic University, 80143 Naples, Italy; stefania.cataldi@unipegaso.it

**Keywords:** wearable technologies, human-centered AI, tele-exercise, digital biomarkers, decision support, functional assessment, remote monitoring

## Abstract

Machine learning (ML) is increasingly influencing the assessment and delivery of movement and exercise, yet its role within adapted physical activity (APA) for individuals with chronic conditions has not been comprehensively synthesized. ML-based approaches have the potential to enhance functional assessment, support individualized exercise prescription, and facilitate scalable monitoring across preventive, community-based, and long-term adapted exercise settings, particularly in populations characterized by functional heterogeneity and variable responses to exercise. The aim of this narrative review is to synthesize and critically discuss current ML applications relevant to the core professional processes of APA practice. A structured narrative review was conducted using searches in PubMed/MEDLINE, Scopus, and Web of Science, complemented by targeted searches in engineering-oriented sources to capture ML methods not consistently indexed in biomedical databases. The search covered the period in which contemporary ML approaches have been increasingly applied to human movement and exercise research and was last updated in January 2026. Evidence was synthesized thematically into application-oriented domains relevant to APA practice. ML applications in APA include markerless motion and gait analysis, wearable-sensor data processing, balance and fall-risk assessment, and functional classification. Predictive and adaptive models support individualized regulation of exercise intensity, progression, and workload, including remote and hybrid delivery models. Applications span oncology, cardiometabolic, respiratory, neuromuscular conditions, and adapted sport contexts. Ethical, legal, and governance issues, such as algorithmic bias, data privacy, and professional accountability, emerge as central considerations for safe and equitable implementation. ML represents a promising decision-support layer for APA, complementing professional expertise through enhanced assessment, personalization, and monitoring. Its effective integration requires robust validation, interpretability, and responsible governance to ensure that ML augments, rather than replaces, professional judgment in APA practice.

## 1. Introduction

Machine learning (ML) has rapidly become a central methodological tool in sports medicine and human movement science, with growing applications in exercise-based assessment, prescription, and monitoring. Over the last decade, ML has been increasingly applied to movement classification, gait and posture analysis, fall risk prediction, injury surveillance, and automated interpretation of wearable sensor data [1,2,3]. By learning complex, often non-linear patterns from large datasets, ML algorithms can extract clinically relevant information from kinematic, kinetic, physiological, and contextual signals that would be difficult or impossible to capture using traditional analytical approaches [1,4]. In parallel, advances in computer vision and deep learning have enabled markerless motion capture and pose estimation from standard video, substantially expanding the feasibility of movement assessment in non-laboratory and real-world settings [5,6].

These developments are highly relevant for adapted physical activity (APA) specialists, kinesiologists, and clinical exercise professionals working across clinically supervised and community-based adapted exercise services, where there is a growing interest in objective, continuous, and ecologically valid measures of physical function. Unlike performance-oriented sport training or device-centered rehabilitation models, APA practice is primarily concerned with long-term functional participation, contextual adaptation, and safe progression across heterogeneous clinical-to-community pathways. Recent work has demonstrated that ML models can classify gait impairments, quantify balance deficits, detect subtle motor changes, and predict clinically meaningful outcomes in conditions such as Parkinson’s disease, stroke, musculoskeletal disorders, and frailty [7,8,9]. Similarly, ML has been used to interpret multimodal data from wearable devices and smartphones, offering new opportunities for remote monitoring, tele-exercise, and digitally supported exercise programs [10,11,12]. Emerging studies have also shown that ML-based models can reliably quantify posture, detect compensatory patterns, and improve the reproducibility of functional assessments [13,14].

Despite this rapid progress, the potential of ML within APA remains only partially addressed in the current literature. APA encompasses structured exercise and motor interventions tailored to individuals living with chronic diseases, disability, or functional limitations, with the aim of improving physical, psychological, and social outcomes in both clinical and community settings [15]. Within this review, APA is conceptualized as the function-oriented and context-adapted application of exercise science, distinct from performance-driven sport training and from purely rehabilitative or device-centered interventions. These populations often present heterogeneous functional capacities, multimorbidity, and variable responses to exercise, which demand ongoing assessment, careful progression, and highly individualized prescription [16,17]. From this perspective, APA represents a particularly suitable domain for ML-based approaches, given their ability to handle high-dimensional data, capture inter-individual variability, and support data-informed decision-making. Moreover, initial evidence suggests that ML can support pilot-level remote-guided and hybrid exercise interventions in chronic disease populations, helping bridge clinical supervision and real-world practice [12,18,19,20].

However, existing literature tends to fragment ML applications across separate domains: sport performance, specific clinical populations, or technology-focused reviews on wearables, exergaming, or remote exercise delivery [3,6,11,19,21,22]. While these reviews provide valuable technical and condition-specific insights, they generally do not integrate ML applications within the core professional processes of APA practice, such as structured functional assessment, individualized FITT-VP prescription, progression management, and safety accountability across clinical-to-community pathways. Moreover, implementation feasibility, professional decision-support integration, and cross-condition synthesis within real-world APA service models remain insufficiently addressed. As a result, no narrative synthesis currently examines ML from an explicitly APA-centered, practice-oriented perspective that bridges technological innovation with professional workflows and organizational contexts.

In this context, a focused narrative synthesis is warranted to bridge recent technological advances in ML with the practical and organizational realities of APA. Therefore, the aims of this review are to: (i) synthesize current evidence on ML applications relevant to functional assessment, individualized exercise prescription, monitoring, and outcome prediction in APA; (ii) illustrate key examples across oncology, metabolic, cardiovascular, respiratory, neuromuscular conditions, and adapted sport; (iii) propose a conceptual framework for integrating ML into APA practice in clinical and community settings; and (iv) discuss ethical, legal, and organizational implications of ML adoption in vulnerable populations.

Given the methodological heterogeneity of ML applications in APA, spanning diverse data modalities, populations, and implementation contexts, a structured narrative review was deemed more appropriate than a formal systematic synthesis. This approach allows for conceptual integration across emerging domains while maintaining transparency in search strategy and thematic organization.

## 2. Materials and Methods

This review adopted a structured narrative review approach, consistent with established methodological guidance for narrative synthesis [23]. A narrative design was selected because research on ML in APA spans heterogeneous domains, including exercise science, biomechanics, wearable sensing, and computer vision, making rigid systematic criteria difficult to operationalize. For example, studies in this area range from proof-of-concept deep-learning models trained on small sensor datasets, to retrospective analyses of wearable-derived physiological signals, to pilot implementations of tele-exercise systems in specific clinical populations. Such methodological variability in data sources, outcome definitions, validation procedures, and implementation settings complicates the application of uniform systematic eligibility criteria and quantitative comparison frameworks. Moreover, the rapid evolution of ML methods and the interdisciplinary nature of the field favor a flexible synthesis capable of integrating methodological, applied, and conceptual evidence. The primary objective was to develop an integrative framework linking ML techniques to functional assessment, exercise prescription, monitoring, and real-world implementation in APA contexts, an aim aligned with the strengths of structured narrative reviews [23]. Consistent with this approach, emphasis was placed on transparency of the search strategy and conceptual coherence rather than exhaustive quantitative comparison.

### 2.1. Literature Search

A structured literature search was conducted in PubMed/MEDLINE, Scopus, and Web of Science between November 2025 and January 2026, identifying studies published between January 2018 and January 2026. To capture relevant methodological contributions originating from engineering and computer science, targeted searches were also performed in IEEE Xplore, ACM Digital Library, and Google Scholar, focusing on studies with clear translational relevance to human movement, exercise, and APA. Translational relevance was operationalized as the inclusion of human participants or human movement datasets, explicit functional or physiological outcome measures, and a clearly articulated link to exercise, rehabilitation, or adapted physical activity contexts. Purely technical algorithm development studies without human application or functional interpretation were excluded. Searches in IEEE Xplore and ACM Digital Library were conducted using structured keyword combinations analogous to those applied in the primary databases, adapted to platform-specific indexing systems and guided by the same inclusion principles, while acknowledging the inherent heterogeneity of engineering-oriented evidence. Search terms combined expressions related to machine learning, exercise, adapted physical activity, and key movement constructs (e.g., gait, posture, balance), together with technology-oriented terms such as wearable sensors, pose estimation, and digital biomarkers. Search strings were iteratively refined to balance sensitivity and specificity, ensuring inclusion of clinically relevant ML applications while excluding purely technical engineering studies without translational relevance. An example search string was: (“machine learning” OR “artificial intelligence”) AND (“adapted physical activity” OR “exercise prescription” OR “functional assessment”). The complete database-specific search strings are provided in Supplementary Table S1 to enhance transparency and reproducibility. The search focused on studies published from 2018 onward, reflecting the period in which contemporary ML approaches, including ensemble and deep learning methods, became methodologically mature and widely applied in human movement and exercise research. Earlier studies were not excluded a priori but were considered outside the primary scope when not aligned with current data-driven and sensor-based ML paradigms.

### 2.2. Study Selection and Thematic Synthesis

Inclusion studies were eligible if they: (1) applied ML or artificial intelligence methods; (2) addressed human movement, exercise, or functional assessment; (3) involved populations or contexts relevant to APA (e.g., chronic diseases, disability, older adults, adapted sport); and (4) provided sufficient methodological or applied detail to allow conceptual interpretation within APA professional processes.

Studies focused exclusively on elite sport performance without relevance to adapted or clinical exercise contexts, engineering papers without human data or functional outcomes, conference abstracts without full texts, and opinion pieces lacking empirical or methodological contributions were excluded. Eligibility criteria were applied during title/abstract screening and subsequently confirmed through full-text assessment to ensure conceptual and contextual relevance to ML-supported APA.

Data extraction emphasized conceptual and functional relevance rather than quantitative outcomes. For each study, information was systematically examined regarding: (i) study design characteristics; (ii) population and clinical context; (iii) data sources and sensing modalities; (iv) ML methodology and validation approach; (v) movement or physiological outcomes assessed; and (vi) the primary application domain (assessment, prescription, monitoring, or implementation). Evidence was synthesized using an application-oriented thematic structure, commonly adopted in interdisciplinary ML reviews, in which conceptual domains emerge from recurring functional use cases rather than formal qualitative coding [1,2]. The synthesis followed a hybrid approach: preliminary macro-domains were defined a priori based on core APA professional processes, while subthemes and cross-condition applications emerged inductively during full-text review. Two authors independently assigned each retained study to the most appropriate domain based on its primary functional use case and intended relevance for APA practice; disagreements were resolved through discussion until consensus, with adjudication by a third author when necessary. Domain definitions were iteratively refined by collapsing overlapping themes and re-checking consistency across the full-text set.

Based on this synthesis, evidence was organized into four recurrent domains: (i) ML-based functional assessment; (ii) ML-supported personalization of exercise prescription; (iii) monitoring, outcome prediction, and tele-exercise applications; and (iv) cross-condition applications spanning oncology, metabolic, cardiovascular, respiratory, neuromuscular conditions, and adapted sport. This framework reflects the operational roles of ML within contemporary APA practice and supports coherent interpretation across heterogeneous clinical and community-based contexts [1,2].

## 3. Results

Results are structured to first describe methodological foundations of ML, followed by its applications in functional assessment, personalized exercise prescription, monitoring and tele-exercise, integration within clinical and community APA practice, and related ethical, legal, and health-system considerations. Across domains, studies varied substantially in the validation stage, ranging from proof-of-concept models tested in controlled datasets to applications evaluated in real-world or clinical settings. Where relevant, distinctions between exploratory and clinically implemented systems are explicitly indicated below.

### 3.1. Foundations of Machine Learning for Movement and Exercise-Based Interventions

Analysis of the selected literature revealed a first overarching thematic domain concerning the foundational principles and methodological approaches of ML as applied to human movement and exercise-based interventions across clinical, preventive, and APA contexts. Across studies, ML techniques were categorized into three broad families, namely supervised learning, unsupervised learning, and deep learning, reflecting widely recognized methodological taxonomies in the machine learning field and used here as an analytical framework to organize the evidence rather than as a novel classification. Table 1 provides a concise comparison of supervised, unsupervised, and deep learning approaches, with APA-oriented examples, typical data sources, and representative practice outputs. Each contributed uniquely to the analysis of motor patterns and functional performance. Supervised learning algorithms, such as support vector machines, random forests, and gradient boosting, were frequently used to classify gait deviations, detect balance impairments, or predict functional outcomes when labeled datasets were available [24,25]. In contrast, unsupervised approaches, including clustering and dimensionality reduction, were applied to uncover latent movement patterns or heterogeneity in motor behaviors without predefined categories, which is particularly relevant for chronic conditions characterized by diverse functional phenotypes [26,27]. Deep learning models, moreover, have gained substantial prominence in the last five years due to their ability to process complex, high-dimensional data from video, inertial sensors, or multimodal physiological streams, enabling markerless pose estimation and automated movement segmentation with high accuracy in exercise, APA, and movement assessment settings [5,6]. Within this methodological landscape, APA emerges as a context in which assessment, prescription, and monitoring are inherently individualized and continuously adapted to functional capacity and variability associated with chronic conditions.

Across the included literature, a second foundational theme concerned the integration of diverse data sources, reflecting the expansion of ML beyond traditional laboratory-based biomechanics. Wearable sensors, including inertial measurement units (IMUs), accelerometers, gyroscopes, and electromyography sensors, were widely used to capture spatiotemporal gait variables, postural oscillations, upper- and lower-limb kinematics, and physiological responses during exercise [2,10]. Complementary to wearables, video-based and computer-vision approaches were increasingly adopted, particularly through deep-learning-based pose estimation frameworks such as OpenPose, MediaPipe, and DeepLabCut, which allow movement assessment without markers or laboratory infrastructure [5,6,28]. Several studies also incorporated clinical or electronic health record datasets, merging functional data with demographic, medical, or behavioral variables to improve the prediction of exercise tolerance, fall risk, or functional outcomes relevant to exercise-based and APA programs [4,11]. Additionally, recent work has shown that ML can enhance reliability and reproducibility in postural assessment, supporting its relevance for both clinical and community-based APA settings [14].

A consistent finding across sources was that ML methods often showed improved sensitivity over traditional analytic techniques in detecting subtle motor impairments, characterizing gait variability, or identifying complex relationships between functional performance and health outcomes. For example, several studies reported classification accuracies for gait impairment or fall-risk detection typically ranging between 80% and 95%, with area-under-the-curve (AUC) values frequently above 0.85 when validated on independent datasets [24,25]. In markerless motion analysis and pose-estimation tasks, deep learning models have demonstrated high agreement with laboratory-based reference systems, often exceeding 85–90% correlation or concordance metrics [5,6]. Traditional biomechanical analyses typically rely on linear models or manually extracted features, whereas ML can model non-linear, multivariate patterns inherent to real-world movement behavior [1]. This advantage is particularly relevant in APA, where individuals with chronic conditions frequently exhibit heterogeneous motor profiles and fluctuating functional capacities that are difficult to capture using conventional approaches. However, performance and generalizability depend strongly on data quality, population representativeness, and external validation, which remain uneven across APA-relevant contexts.

Finally, the literature emphasized the growing role of big data in human movement science. Advances in wearable technology, mobile health applications, tele-exercise platforms, and home-based monitoring systems have generated unprecedented volumes of continuous movement and physiological data. ML algorithms are uniquely positioned to exploit these datasets, enabling scalable, ecologically valid assessments and supporting individualized decision-making along the continuum of exercise-based care, ranging from supervised clinical exercise to community-based APA programs and long-term functional maintenance [7,11]. This data-driven perspective provides the conceptual foundation for subsequent thematic domains concerning functional assessment, personalized exercise prescription, and monitoring.

### 3.2. Machine Learning for Functional Assessment in Adapted Physical Activity (APA)

Across the reviewed literature, a prominent thematic domain concerned the use of ML techniques to enhance functional assessment in populations relevant to APA. This domain reflects the rapid shift from traditional laboratory-based evaluations toward automated, scalable, and ecologically valid approaches capable of quantifying movement in clinical, community, and everyday environments.

#### 3.2.1. Pose Estimation and Markerless Motion Analysis

A substantial body of work demonstrated that deep-learning–based pose estimation systems such as OpenPose, MediaPipe, and DeepLabCut allow accurate extraction of joint kinematics from standard video recordings, without the need for reflective markers or specialized equipment. These systems have been successfully applied to estimate gait parameters, detect compensatory movement strategies, quantify balance responses, and evaluate motor function in populations with chronic conditions [5,28,29]. Markerless motion capture is particularly relevant for APA, as it enables professionals to conduct movement assessments in community settings, such as gyms, rehabilitation centers, or home environments, expanding access beyond biomechanics laboratories. Pose-estimation pipelines have shown promising validity for step detection, joint angle estimation, sit-to-stand analysis, and basic functional tests commonly used in adapted exercise programs [6,29,30]. In validation studies comparing markerless systems with laboratory-based motion capture, joint-angle estimation errors have typically ranged between approximately 3–8° under controlled conditions, with intraclass correlation coefficients frequently exceeding 0.80. In more ecologically valid or home-based settings, error margins may increase modestly (often within 5–10°), but agreement generally remains within ranges considered acceptable for functional screening and progression monitoring in APA contexts [5,6].

#### 3.2.2. Automated Gait Analysis

ML techniques were extensively applied to gait analysis, a core component of functional evaluation in many chronic conditions. Studies using wearable inertial measurement units (IMUs) consistently demonstrated that supervised algorithms (e.g., random forests, convolutional neural networks) can classify gait abnormalities, distinguish pathological patterns, and identify subtle deviations associated with neurological, metabolic, and cardiovascular conditions [8,9]. Deep learning models trained on spatiotemporal gait features have reported classification accuracies typically ranging between approximately 85% and 95%, with area-under-the-curve (AUC) values frequently above 0.85 in controlled or condition-specific datasets when detecting freezing-of-gait episodes in Parkinson’s disease, impaired gait symmetry in stroke survivors, or reduced gait stability in older adults [7,21]. However, most of these performance metrics derive from internally validated or laboratory-based datasets with relatively homogeneous samples. Evidence from externally validated, community-based, or real-world APA deployments remains more limited, and model performance may decrease when applied to heterogeneous populations, variable walking speeds, or unconstrained environmental conditions. Recent biomechanical evidence also highlights the importance of natural gait variability for ML-based classification, supporting movement assessments performed at self-selected walking speeds [14]. These approaches support APA professionals in screening functional limitations, monitoring progression, and tailoring exercise programs to individual gait profiles.

#### 3.2.3. Posture, Balance, and Fall-Risk Assessment

ML applications targeting posture and balance highlight another area of relevance to APA. Neural networks trained on center-of-pressure trajectories, accelerometry signals, or video-based sway measures have demonstrated strong performance in identifying balance impairments, predicting fall risk, and distinguishing between functional severity levels [31,32]. Combining wearable sensors with ML has proven effective in quantifying postural transitions, trunk control, and reactive balance responses during functional tasks, outcomes highly relevant for exercise prescription in older adults and individuals with chronic conditions [32]. Importantly, ML-based balance metrics often outperform traditional threshold-based measures, providing richer and more individualized information [31]. Emerging ML-based approaches for posture analysis further support APA applications by improving reliability and reducing assessor-related variability, especially in non-laboratory settings [14].

#### 3.2.4. Wearable Sensors and Neural Models for Multimodal Functional Data

A recurring theme was the integration of multimodal wearable-sensor data, including accelerometers, gyroscopes, magnetometers, heart-rate sensors, and respiratory inductance signals, into ML models capable of detecting functional impairments and estimating exercise capacity. Multisensor fusion techniques enhanced the detection of gait variability, upper-limb kinematics, exertion levels, and movement quality during everyday activities or structured exercise [2,11]. Recurrent neural networks and convolutional architectures trained on time-series data have been particularly effective in capturing dynamic movement characteristics relevant to APA interventions [11].

#### 3.2.5. Functional Classification and Risk Stratification

Several studies applied ML for functional classification, identifying subgroups of individuals with similar movement patterns or functional limitations. Clustering methods revealed distinct mobility phenotypes in older adults, neurological populations, and individuals with balance impairments [33]. Supervised models have been used to stratify fall risk, categorize gait impairment severity, or classify functional performance levels relevant to adapted exercise prescription [4,31]. These methods support APA professionals by enabling more accurate functional profiling, targeted interventions, and dynamic adjustment of exercise programs based on predicted risk or expected responsiveness.

- Summary of Key Points

Together, these findings highlight the expanding role of ML as a powerful tool for comprehensive and individualized functional assessment in APA. Across pose estimation, gait analysis, balance and posture evaluation, multimodal wearable sensing, and functional classification, ML frameworks enable objective quantification of movement patterns, improved detection of functional impairments, and more refined risk stratification. By leveraging video-based approaches, wearable sensors, and integrated data streams, ML has the potential to democratize movement analysis and support scalable functional assessment across clinical, community, and home environments, which is a central requirement for contemporary APA practice. Most functional-assessment applications are currently supported by proof-of-concept or internally validated studies, with more limited evidence from externally validated and real-world APA deployments.

For clarity and synthesis, Table 2 summarizes the main ML application domains relevant to functional assessment in APA, including data sources, analytical approaches, practice-oriented outcomes, and indicative study design characteristics, typical validation metrics, validation context (laboratory, clinical, or community-based), and approximate sample-size ranges to inform implementation readiness.

### 3.3. Machine Learning for Personalized Exercise Prescription

A second major thematic domain emerging from the literature concerned the use of ML to enhance personalized exercise prescription, a core component of APA. Across studies, ML techniques were employed to model individual responses to exercise, optimize training variables, and support dynamic prescription adjustments tailored to functional and clinical characteristics.

#### 3.3.1. Predictive Models for Intensity, Volume, Modality, and Progression

Several publications demonstrated the potential of ML to develop predictive models capable of estimating optimal exercise intensity, volume, modality, or weekly progression for individuals with heterogeneous functional profiles. Supervised algorithms, such as random forests, gradient boosting machines, and support vector regressors, have been used to predict aerobic capacity, muscular strength gains, and cardiometabolic responses based on demographic features, wearable data, and baseline functional assessments [34,35]. In populations with chronic disease, ML-based predictive models have also been applied to estimate tolerance to exercise sessions, identify risk thresholds, and forecast changes in movement efficiency or fatigue over time [36].

Recent work using deep learning and physiological data from wearable sensors has demonstrated the feasibility of predicting exercise intensity levels and exertion patterns through recurrent neural networks, supporting personalized and real-time exercise regulation models [37]. In chronic disease populations, evidence from adapted and multicomponent exercise interventions highlights marked inter-individual variability in clinical and functional responses to training, reinforcing the need for individualized progression strategies that predictive ML approaches aim to address [22].

These predictive capabilities provide an evidence-driven foundation for adjusting traditional FITT-VP variables to individual needs. However, most predictive models remain population- or condition-specific and have been validated primarily within controlled or single-cohort contexts. External validation across diverse clinical and community-based APA settings remains limited, and routine integration into prescription workflows therefore requires further multi-site and real-world testing.

#### 3.3.2. Automated Identification of Exercise Response Profiles

Another recurring application of ML involved the automatic detection of individual response patterns. Clustering and unsupervised learning techniques have identified distinct phenotypes of exercise responsiveness, such as high, moderate, or low responders, based on physiological, biomechanical, or behavioral data [38]. Similarly, neural-network–based classifiers have differentiated between adaptive and maladaptive responses to endurance or resistance training, capturing subtle changes in heart-rate variability, movement quality, or autonomic function [37]. This automated stratification aligns closely with APA needs, where functional heterogeneity is the rule rather than the exception, and early prediction of responsiveness can optimize intervention planning.

#### 3.3.3. Personalization for Individuals with Chronic Conditions

In individuals with chronic diseases, including cancer survivors, patients with metabolic or cardiovascular disorders, respiratory impairments, or neuromuscular conditions, ML-driven personalization has been used to identify functional constraints, comorbidity interactions, and safety considerations that traditional linear models struggle to capture. Studies across oncology populations have employed ML to predict fatigue trajectories, exercise tolerance, and risk of functional decline. Beenhakker et al. [39] developed ML models to predict long-term cancer-related fatigue in breast cancer survivors, achieving moderate discriminative performance (C-statistic ~0.67) and highlighting the importance of patient-reported and treatment-related features. Wang et al. [40] validated predictive models for cancer-related fatigue in lymphoma survivors, where pain, physical function, and sleep disturbance emerged as key predictors.

ML-based functional modeling has also supported individualized progression in APA programs for cancer survivors, particularly in contexts where fatigue and deconditioning fluctuate substantially across training cycles. In cardiometabolic conditions, ML models using wearable-derived physiological data have effectively estimated cardiorespiratory fitness during free-living activities. Frade et al. [41] employed support vector regression on week-long wearable data to estimate VO_2_max, achieving accurate prediction without requiring formal exercise tests. For respiratory or neuromuscular conditions, digital health and remote monitoring technologies facilitate adaptation of workload to daily symptom variability. A systematic review by Klein et al. [42] examined remote videoconferencing-based fitness assessments across diverse clinical settings, underscoring the feasibility of dynamically monitoring strength, endurance, and balance, foundational steps toward responsive, ML-informed workload adjustment.

#### 3.3.4. Real-Time Adaptations: Closed Loop and Adaptive Systems

A consistent finding across emerging technological approaches is the potential of machine learning (ML) to support real-time adaptation of exercise prescriptions through closed-loop and adaptive systems. These frameworks integrate continuous sensor-derived data, such as heart rate dynamics, respiratory patterns, and movement quality, with ML models to enable dynamic regulation of exercise parameters, including cadence, intensity, or workload, based on immediate physiological feedback [36,37]. In tele-exercise and remote APA programs, adaptive ML-based systems have been shown to detect early signs of fatigue, coordination loss, or excessive exertion, enabling automated workload adjustments or alerts to supervising professionals [16,21]. Such adaptive monitoring approaches are particularly relevant in decentralized exercise delivery models, where continuous in-person supervision is not feasible.

Closed-loop ML systems are especially pertinent for APA practice because they can accommodate high intra-individual variability, which is common in oncology, metabolic syndrome, heart failure, chronic respiratory disease, Parkinson’s disease, and post-stroke populations. By dynamically adjusting exercise demands in response to real-time physiological and functional signals, these systems support safer intensity modulation and personalized progression across heterogeneous chronic conditions [36,37]. Overall, closed-loop and adaptive ML frameworks represent an important step toward precision exercise interventions that continuously respond to intra-individual variability, bridging predictive modeling with real-world exercise implementation in clinical and community-based APA settings [21]. While conceptually promising, current closed-loop ML systems for APA have been validated predominantly in controlled laboratory or supervised clinical settings. Evidence from large-scale, real-world, and unsupervised community-based implementations remains limited, and further pragmatic validation studies are needed to establish safety, reliability, and long-term feasibility in routine APA practice.

#### 3.3.5. Structured Integration with Traditional FITT-VP Principles

Finally, the literature demonstrates growing interest in integrating ML-derived insights with the conventional FITT-VP framework (Frequency, Intensity, Time, Type, Volume, Progression), particularly within chronic disease and remote exercise contexts. Rather than replacing FITT-VP, ML approaches provide a data-driven augmentation that supports more precise regulation of training variables, such as intensity ranges, workload progression, and session volume, based on physiological and behavioral data collected during exercise [16,36,37]. For example, ML detection of elevated fatigue markers, reduced heart-rate variability, or deteriorating movement quality may prompt temporary adjustment of Frequency, Intensity, or Time (e.g., reducing weekly sessions from three to two; lowering from 60% to 50% of predicted maximal capacity; shortening session duration from 45 to 30 min) to prevent overload, whereas consistent improvements may support controlled progression of volume and workload [16,21,36,37].

ML-enabled personalization aligns with the broader shift toward precision and adaptive exercise models, supporting APA professionals in balancing standardized exercise guidelines with individual variability in functional capacity, symptom burden, and response to training [21]. This integration is particularly valuable for conditions characterized by fluctuating symptom patterns, such as cancer-related fatigue, autonomic instability in Parkinson’s disease, or dyspnea variability in chronic respiratory disorders, where ML-supported monitoring can inform when FITT-VP prescriptions should be adapted dynamically rather than applied as fixed protocols [8,16,36,39].

- Summary of Key Points

Collectively, these findings indicate that ML-based approaches can substantially enhance personalized exercise prescription in APA by supporting predictive modeling, response profiling, and real-time workload adaptation. Across chronic conditions, ML enables more precise regulation of FITT-VP variables by accounting for inter- and intra-individual variability in functional capacity, symptom burden, and physiological responses to exercise. These approaches provide a scalable framework for safer and more responsive exercise prescription in both clinical and community-based APA programs. Evidence for ML-supported personalization, particularly real-time closed-loop prescription, remains largely based on pilot studies and internal validation, with limited externally validated trials and scarce long-term implementation data in routine APA practice.

### 3.4. Machine Learning Across Chronic Conditions

In the context of this review, condition-specific evidence is discussed insofar as it informs APA-relevant processes (functional assessment, prescription, monitoring) across clinical-to-community exercise pathways. Across conditions, translation to routine APA practice is constrained by heterogeneous datasets, limited external validation, and variable reporting of deployment details. For consistency across conditions, the following subsections summarize evidence in terms of (i) ML-enabled assessment applications, (ii) prescription/progression support, (iii) monitoring and safety-related functions, and (iv) indicative implementation readiness in APA pathways.

#### 3.4.1. Oncology

Within oncology populations, ML applications have expanded substantially, reflecting the complexity and heterogeneity of functional impairments experienced across the cancer continuum [39,40]. The reviewed literature identified several thematic areas in which ML contributes to enhanced assessment, monitoring, and personalization of APA programs for cancer survivors.

- Functional impairment detection and movement analysis

Cancer-related fatigue, neuropathy, sarcopenia, and treatment-induced motor impairments often produce subtle alterations in gait, balance, and movement quality [30]. ML-enabled gait analysis and wearable-sensor data interpretation have been used to quantify functional decline and detect early deviations that may not be apparent through standard clinical assessments. Supervised models trained on accelerometry or IMU data have differentiated between cancer survivors with high versus low fatigue burden, identified compensatory gait strategies following chemotherapy, and estimated real-world mobility patterns associated with reduced functional capacity [39,40]. Deep-learning–based pose estimation has also been applied to video recordings of functional tasks, such as sit-to-stand, stair climbing, and short walking bouts, to automatically extract kinematic parameters relevant to APA decision-making [6,29]. Additionally, preliminary functional oncology data suggest that ML-based movement signatures may help characterize fatigue-related motor inefficiencies and adaptive movement patterns [21]. These functional movement signatures provide upstream indicators that can be integrated with symptom and contextual data to inform subsequent prediction tasks, particularly fatigue trajectories and exercise tolerance.

- Prediction of fatigue, physical decline, and exercise tolerance

ML models have been increasingly used to forecast fluctuations in cancer-related fatigue, one of the central determinants of exercise adherence and progression. Predictive algorithms integrating physiological, behavioral, and wearable-derived markers have shown the ability to anticipate fatigue trajectories during exercise programs, enabling professionals to adjust session intensity or rest intervals based on expected tolerance [40]. Similar approaches have been used to predict functional decline associated with sarcopenia or cachexia, often integrating gait variability metrics, activity-monitoring features, and self-reported symptoms [43,44]. Emerging APA-focused studies also indicate that ML can support early identification of individuals at risk of low engagement or poor tolerance during multicomponent programs, offering a valuable tool for refining progression in cancer survivors [21]. In practical APA workflows, these forecasts translate into actionable monitoring needs, i.e., identifying when closer supervision, adaptive pacing, or recovery-focused sessions may be required during periods of predicted low tolerance.

- Monitoring exercise responses using multimodal data

ML-enabled multimodal monitoring (e.g., heart rate variability, movement quality, step dynamics, rate of perceived exertion estimation) has shown promise in capturing individualized responses to exercise among cancer survivors. Studies using wearables combined with neural-network classifiers have detected early signs of excessive exertion, compensatory movement patterns indicating neuromuscular fatigue, or reductions in step regularity associated with transient symptom exacerbation [11,34]. Markerless posture and movement analytics, validated in general populations [6], may further support oncology APA programs by improving detection of compensatory strategies typical of fatigue or neuropathy. These tools are particularly relevant for APA programs conducted in community or home environments, where real-time professional supervision may be limited.

- Stratification and personalization of APA programs

Another emerging application involves functional stratification and personalized prescription. Unsupervised learning methods have been used to identify subgroups of cancer survivors with similar movement characteristics or fatigue–mobility profiles, providing a data-driven basis for individualized exercise programming [27]. Predictive models have also been used to estimate adherence likelihood, drop-out risk, and expected improvement trajectories, supporting tailored FITT-VP planning, including adjustments to intensity, session frequency, or progression rates, based on predicted responsiveness to training [35,36]. Functional clustering models have also been proposed to support decision-making in progressive APA pathways, aligning ML-based stratification with the clinical realities of oncology rehabilitation and exercise oncology practice [21].

- Potential for remote, hybrid, and tele-APA models

ML has also played a key role in supporting the transition toward remote and hybrid APA interventions. Tele-exercise platforms integrating ML-based movement analysis or adaptive feedback have shown promise in maintaining training quality outside supervised environments. These systems can detect unsafe movement compensations, inadequate intensity, or signs of instability during home-based sessions, automatically prompting corrective cues or alerting the professional when necessary [45,46]. Such models expand accessibility for cancer survivors who face logistical, geographical, or fatigue-related barriers to in-person participation.

- Summary of Key Points

Together, the reviewed evidence indicates that ML applications in oncology enable a more nuanced and responsive approach to APA across the cancer continuum. By integrating gait analysis, wearable-derived physiological signals, and multimodal functional data, ML supports early detection of fatigue-related impairments, prediction of exercise tolerance, and monitoring of individualized responses to training. These approaches facilitate functional stratification and personalized adjustment of APA programs, particularly in the presence of fluctuating symptoms such as cancer-related fatigue, deconditioning, or treatment-induced motor deficits. Overall, ML-based tools complement professional expertise by providing continuous, real-world functional insights that support safer progression, improved adherence, and long-term engagement in APA among cancer survivors. However, most oncology-focused ML applications remain supported by small-scale or internally validated studies, with limited large-scale prospective validation in routine APA oncology programs.

#### 3.4.2. Metabolic and Obesity-Related Conditions

ML applications in metabolic disorders and obesity revealed a distinct thematic domain centered on the prediction of exercise tolerance, cardiometabolic responses, and movement abnormalities associated with excess adiposity, insulin resistance, and metabolic dysregulation [35,37,41]. Given the high prevalence of heterogeneity in physical function within these populations, ML techniques have been used to improve characterization of mobility impairments, stratify functional risk, and support individualized planning within APA programs.

- Gait, mobility, and functional patterns in metabolic conditions

Excess body mass and metabolic dysfunction frequently alter gait mechanics, balance control, and movement efficiency. ML models trained on wearable-sensor data have differentiated gait profiles associated with obesity severity, impaired metabolic health, or low functional fitness [10]. Neural networks applied to accelerometry time series have identified deviations in step regularity, trunk stability, and lower limb loading patterns, providing objective metrics to guide adapted exercise prescription. Several studies showed that ML-based gait classification outperformed traditional threshold-based analyses in detecting early functional decline in populations with abnormal or impaired gait patterns relevant to metabolic and obesity-related conditions [24,47]. Evidence from sensor-based ergonomic assessments also supports the capacity of ML to detect altered trunk mechanics and loading patterns in individuals exposed to increased mechanical demand, offering relevant methodological parallels for APA management in obesity [14].

- Prediction of cardiometabolic responses to exercise

ML has gained relevance in estimating cardiorespiratory and metabolic responses during exercise, a key element for tailoring intensity and progression in APA. Predictive algorithms using physiological, anthropometric, and wearable-derived data have been used to estimate VO_2_ responses, ventilatory thresholds, metabolic equivalent levels, and glycemic variability during and after exercise [35,41]. These models offer a data-driven approach to determining individualized training zones, particularly useful in people with obesity, type 2 diabetes, or metabolic syndrome, where conventional intensity prescriptions may be inaccurate due to autonomic dysfunction, medication effects, or variability in aerobic fitness.

- Glycemic control and daily variability

In individuals with type 2 diabetes or insulin resistance, ML models integrating continuous glucose monitoring (CGM) data with activity patterns and heart-rate metrics have demonstrated strong predictive capacity for post-exercise glycemic responses. For example, transfer-learning decision-tree and neural-network models accurately anticipated postprandial glucose fluctuations by combining CGM, exercise effort, and dietary intake data [48]. Such insights are highly relevant for APA programs seeking to optimize metabolic outcomes while accounting for intra-individual variability. Recent diabetes-focused work has shown that ML can also predict fluctuations in perceived exertion and adherence-related behaviors in type 2 diabetes patients, which may support progression planning in individuals with metabolic dysregulation [49]. From an APA standpoint, short-horizon CGM-based predictions can support same-day or next-day adjustments such as shifting session timing relative to meals, selecting lower-glycemic-risk modalities (e.g., lower-impact continuous work vs. high-intensity intervals), and temporarily moderating intensity/volume when elevated dysglycemia risk is anticipated.

- Risk stratification and safety monitoring

Obesity and metabolic diseases are often accompanied by functional limitations, cardiovascular risk factors, and reduced exercise tolerance (Frade et al. [41]). ML-based risk stratification models have been developed to detect abnormal exertion responses, excessive fatigue, or early signs of cardiometabolic instability during exercise [35,36]. These systems support APA professionals by providing individualized safety thresholds, workload predictions, and alerts when sensor patterns deviate from expected norms, an especially valuable feature in community and home-based exercise programs. ML-informed stratification also aligns with APA needs by enabling early identification of individuals requiring lower-impact modalities or modified workloads due to joint stress, dyspnea, or reduced neuromuscular control, particularly in older and multimorbid profiles where fall-risk monitoring is also relevant [31].

- Personalized FITT-VP adaptation

Integrating these insights, ML has been proposed as a tool for personalizing FITT-VP variables in metabolic populations. Neural-network and ensemble models have been used to predict optimal exercise frequency, session duration, and progression rates for improving functional capacity, glycemic regulation, and body composition [50]. In obesity, ML-guided personalization has also been applied to identify individuals more likely to benefit from low-impact or multicomponent programs due to joint stress, reduced balance, or lower limb fatigue. This approach is consistent with emerging APA research demonstrating the need for individualized progression curves in populations with multimorbidity, variable fatigue resistance, or altered motor efficiency [51].

- Summary of Key Points

Overall, the evidence suggests that ML can support metabolic and obesity-related APA programs by improving functional profiling (including gait and movement quality), predicting cardiometabolic and glycemic responses, and enabling safer workload regulation through risk stratification. By integrating multimodal wearable data, CGM-based models, and predictive approaches for exertion and adherence, ML provides a practical foundation for individualized FITT-VP adaptation and responsive progression planning in heterogeneous and multimorbid populations. However, many metabolic-focused ML models remain validated on condition-specific or retrospective datasets, with limited prospective validation in structured APA interventions.

#### 3.4.3. Cardiovascular Conditions

ML applications in cardiovascular diseases (CVD), including coronary artery disease, heart failure, atrial fibrillation, and hypertension, revealed several thematic domains relating to functional assessment, prediction of hemodynamic responses, safety monitoring, and individualized exercise prescription. Given the relevance of exercise-based interventions in cardiovascular prevention and rehabilitation, ML has emerged as a tool capable of identifying nuanced physiological patterns that inform APA programming [52,53,54].

- Movement analysis and functional capacity assessment

ML-enabled analysis of gait, activity patterns, and functional capacity is increasingly used to complement traditional cardiovascular assessment. Wearable IMU data analyzed with supervised algorithms have been shown to classify reduced walking efficiency, impaired cadence regulation, and early mobility decline in individuals with heart failure or ischemic heart disease [55]. Deep-learning–based models have been applied to estimate exercise tolerance and functional capacity from daily activity and submaximal performance data, offering continuous, real-world indicators relevant to APA progression [53]. Additionally, heart-rate variability (HRV) features combined with ML classifiers have demonstrated sensitivity in detecting autonomic dysfunction and reduced exercise tolerance in CVD populations [37]. Recent findings from APA-oriented research also highlight that ML can detect early deviations in movement efficiency and symptom-driven pacing strategies, supporting more precise tailoring of workloads in individuals with chronic cardiovascular limitations [54].

- Prediction of cardiovascular responses to exercise

ML methods have shown strong performance in predicting hemodynamic responses, including heart rate, blood pressure, VO_2_ kinetics, and ventilatory thresholds, during exercise. Gradient boosting and recurrent neural networks trained on wearable physiological signals have been used to anticipate abnormal blood-pressure responses or excessive cardiac load during submaximal exercise, thereby supporting safer prescription strategies [56]. Other studies have demonstrated that ML can estimate ventilatory thresholds without laboratory gas analysis, using wearable respiratory sensors and deep-learning models [57]. Such predictive capabilities are especially relevant for APA professionals who must navigate daily fluctuations in symptoms, medication effects, and autonomic instability in CVD populations.

- Risk stratification and safety monitoring during exercise

A consistent finding across cardiovascular literature is the use of ML to support risk stratification, particularly regarding arrhythmias and exertion-related instability. Wearable ECG data analyzed with convolutional neural networks have achieved high accuracy in detecting atrial fibrillation, premature ventricular contractions, and ischemic patterns during daily activity or light-to-moderate exercise [53]. ML-based alert systems have also been used to identify atypical HR recovery dynamics, disproportionate exertion, or early signs of decompensation in individuals with heart failure, leveraging patient-reported vitals and electronic health records [58]. These approaches align with APA frameworks emphasizing continuous safety monitoring, particularly during higher-risk activities such as interval training, resistance exercise, or remote home-based sessions. However, although these alert systems show strong detection performance, evidence that they reduce exercise-related adverse events in real-world APA delivery remains limited, and benefits are currently inferred mainly from controlled validation and feasibility deployments.

- Personalized exercise prescription for cardiovascular conditions

ML-informed personalization of aerobic and resistance training has been explored as a means to optimize safety and effectiveness in individuals with CVD. Predictive models incorporating demographic, physiological, and wearable-derived features have been used to determine individualized intensity ranges, optimal progression rates, and tolerance thresholds [53,54]. In heart-failure populations, neural-network models have estimated daily variability in exercise readiness, allowing more flexible adjustment of frequency and volume, particularly valuable for APA programs that must accommodate day-to-day symptom variability rather than fixed training prescriptions [53]. Emerging approaches also leverage ML to predict ventilatory thresholds and cardiovascular responses during exercise, supporting dynamic tailoring of training loads [56,57].

- Support for hybrid and tele-APA models in CVD

The adoption of tele-exercise and remote APA programs has accelerated interest in ML-based monitoring systems for cardiovascular populations. Studies integrating wearable sensors, such as IMUs, ECG patches, PPG, and blood pressure devices, with ML algorithms demonstrated the feasibility of real-time supervision, early detection of unsafe physiological responses, and automated adjustment of exercise parameters in remote settings. For instance, wearable sensors have been successfully used to monitor physical activity in heart failure clinical trials, enabling supervised intensity control and safety alerts through ML-driven analyses [55]. Remote fitness assessments via videoconferencing supported by ML analytics have shown potential for delivering tailored cardiovascular exercise and tracking adherence while ensuring that safety thresholds are maintained [42]. Additionally, ML-enhanced wearable blood pressure sensors facilitate continuous estimation of circulatory stress, allowing for dynamic adjustments in exercise load during remote sessions [56]. These systems enhance accessibility for individuals with transportation barriers or limited access to supervised centers and provide APA professionals with scalable tools for delivering personalized cardiovascular exercise with maintained safety.

- Summary of Key Points

Across cardiovascular conditions, ML provides a robust framework for enhancing functional assessment, predicting individualized hemodynamic responses, and supporting safety-oriented exercise prescription within APA programs. By integrating wearable-derived physiological signals, movement data, and patient-reported information, ML enables refined risk stratification, early detection of abnormal exercise responses, and dynamic adjustment of training intensity and volume. These capabilities are particularly relevant in populations characterized by symptom variability, medication effects, and autonomic instability. Furthermore, ML-supported monitoring systems facilitate the safe delivery of hybrid and tele-APA models, helping bridge clinical rehabilitation and community-based exercise pathways while promoting long-term adherence and participation in individuals with cardiovascular disease. Although predictive performance metrics are frequently strong, external validation across diverse clinical and community APA settings remains uneven.

#### 3.4.4. Respiratory Conditions

ML applications in respiratory diseases, particularly chronic obstructive pulmonary disease (COPD), asthma, interstitial lung disease, and conditions characterized by exercise-induced ventilatory limitations, revealed several thematic areas relevant to APA. The reviewed literature highlights ML as a tool for improving functional assessment, predicting ventilatory responses, monitoring exertional tolerance, and supporting individualized exercise programming in populations with compromised pulmonary function [57,59,60].

- Movement patterns, gait changes, and functional limitations

Respiratory diseases are often associated with dyspnea, reduced walking efficiency, altered gait mechanics, and decreased lower-limb endurance. ML models trained on wearable inertial-measurement-unit (IMU) data have successfully identified movement irregularities linked to ventilatory impairment, such as shortened stride length, reduced cadence stability, and compensatory trunk movements [61]. These features have been used to classify disease severity and detect early functional decline, which is essential for tailoring APA interventions. In COPD populations, ML approaches, including clustering and predictive algorithms, have revealed distinct mobility phenotypes characterized by varying levels of activity tolerance, gait variability, and exertional fatigue [62]. Methodological work in ML-based biomechanical analysis also suggests that subtle postural adjustments and thoraco-abdominal compensations, typical in COPD and ILD, may be detectable through sensor-supported movement models [61], providing an additional layer of functional insight for APA professionals.

- Prediction of ventilatory and exertional responses

ML has demonstrated strong potential in predicting ventilatory responses to exercise, including tidal volume, respiratory frequency, blood oxygen trends, and dyspnea levels. Recurrent neural networks and gradient boosting models leveraging wearable sensor data, such as SpO_2_, respiratory inductance plethysmography, and heart-rate dynamics, have accurately forecasted exertional desaturation and symptoms during exercise tasks [57]. Such predictive capabilities inform safe intensity prescription and help prevent symptom exacerbation during APA sessions. In chronic respiratory disease populations, ML models have been used to predict acute respiratory failure, ventilator dependence, and mortality using physiological and exercise-derived features [60], supporting risk stratification and readiness assessment within exercise-based APA programs. Additionally, wide-ranging reviews of wearable-ML interventions in COPD have underscored the efficacy of symptoms and vital-sign prediction for preventing exercise-induced deterioration [59]. These predictive insights are particularly relevant for APA specialists, who must integrate ventilatory limitations with the progression logic of multicomponent or interval-based exercise.

- Monitoring of real-world exertion, dyspnea, and instability

ML-enabled monitoring systems enhance the detection of unsafe exertional patterns, including premature fatigue, ventilatory instability, and muscle deconditioning. Studies combining movement sensors with physiological monitoring have identified patterns associated with imminent dyspnea escalation or inefficient breathing mechanics during daily activity or structured exercise [57,59]. Deep-learning–based anomaly detection has been used to identify abrupt deviations in respiratory rhythm, providing the basis for automated alerts in tele-APA programs for individuals with limited exercise tolerance. Such monitoring approaches complement APA principles, which require fine-tuned adjustments of intensity and cadence based on real-time ventilatory constraints.

- Functional stratification and personalized prescription

Functional stratification using ML has shown value in identifying subgroups of respiratory patients with similar activity profiles, ventilatory limitations, or exercise tolerance patterns [62]. These data-driven classifications support the personalization of FITT-VP variables. Predictive models have been used to estimate safe training intensities, adjust walking cadence or interval structure, and anticipate days of lower functional capacity due to symptom fluctuation or environmental triggers [57,60]. ML-based personalization aligns well with APA needs in COPD, ILD, and severe asthma, where daily variability in dyspnea, fatigue, and ventilatory efficiency is often substantial. Recent reviews also emphasize that wearable-ML systems in COPD can support individualized progression strategies and remote monitoring [59].

- Support for remote and hybrid APA programs

Tele-APA and home-based exercise programs for respiratory populations increasingly integrate ML algorithms for real-time safety monitoring. Systems incorporating wearable SpO_2_ sensors, motion data, and respiratory signals have been used to detect exertional desaturation, poor ventilatory efficiency, and early signs of exacerbation, enabling remote supervision and adaptive exercise recommendations [57,59]. ML-based feedback can help participants maintain safe exercise intensity without continuous face-to-face supervision. Predictive models for respiratory failure and ventilatory instability further support individualized safety thresholds in remote programs [60]. These technologies are particularly valuable in respiratory conditions, where barriers such as dyspnea, fatigue, mobility limitations, and geographic constraints often reduce access to supervised in-person programs.

- Summary of Key Points

Overall, the literature indicates that ML plays a crucial role in enhancing functional assessment, predicting ventilatory and exertional responses, and supporting safety-oriented exercise prescription in individuals with chronic respiratory conditions. By integrating wearable-derived movement, ventilatory, and physiological data, ML enables refined detection of exertional limitations, dyspnea escalation, and ventilatory instability, which are central determinants of exercise tolerance in APA. ML-based functional stratification and predictive modeling further support individualized adjustment of FITT-VP variables in the presence of high day-to-day symptom variability. Importantly, these capabilities facilitate the safe implementation of remote and hybrid APA programs, expanding access to supervised exercise while maintaining individualized monitoring and progression in populations with compromised pulmonary function. Despite promising predictive accuracy, most respiratory-focused ML applications require further external validation and standardized reporting before routine integration into APA practice.

#### 3.4.5. Neuromuscular Disorders

ML applications in neuromuscular disorders, including Parkinson’s disease, multiple sclerosis, amyotrophic lateral sclerosis (ALS), peripheral neuropathies, muscular dystrophies, stroke-related motor impairment, and cerebral palsy, highlighted a broad thematic domain focused on movement analysis, functional classification, fatigue and symptom prediction, and individualized adaptation of exercise programs [63]. These populations exhibit heterogeneous motor deficits, fluctuating symptom severity, and complex compensatory strategies, making ML particularly useful for supporting exercise-based APA interventions across clinical, community, and home-based settings.

- Movement analysis and detection of motor abnormalities

ML applied to wearable sensors, surface EMG, and markerless motion capture has demonstrated high accuracy in detecting neuromuscular impairments such as bradykinesia, tremor, rigidity, gait asymmetry, reduced stride length, and abnormal postural transitions [8,25]. Deep learning approaches trained on inertial data have been used to segment functional tasks (e.g., sit-to-stand, turning, reaching) and quantify deviations relevant for APA progression (Gu et al. [63]). In stroke populations, convolutional networks have distinguished between compensatory versus efficient gait phases, supporting targeted training decisions [64]. ML-based postural and biomechanical assessment models, validated in healthy populations as methodological benchmarks rather than clinical tools [14], offer methodological insight for detecting subtle compensations and alignment deviations that frequently characterize neuromuscular disorders.

- Functional capacity, fatigue, and symptom prediction

A second thematic area involved ML-based prediction of symptom fluctuations and fatigue, key elements influencing exercise readiness and progression. ML models using time-series data from wearable sensors have predicted freezing-of-gait risk and motor instability in Parkinson’s disease, supporting individualized exercise planning [65]. In multiple sclerosis, ML approaches integrating imaging biomarkers and clinical features have been applied to predict disease progression and mobility decline, which can inform fatigue management during APA programs [66]. These approaches align with APA needs, where individualized progression is essential due to fluctuating neuromuscular function. Preliminary evidence suggests that ML-based wearable systems can support personalized progression strategies and functional monitoring in rehabilitation contexts [63].

- Classification of functional phenotypes

Unsupervised ML approaches have revealed distinct functional phenotypes, operationalized as clusters of movement patterns and functional profiles identified through unsupervised ML approaches, across neuromuscular populations, including gait asymmetries, postural control deficits, and compensatory strategies [63,67]. In progressive neuromuscular disorders, ML-based classification of upper- and lower-limb performance supports early detection of functional decline and informs adapted exercise interventions [63]. For stroke survivors, deep-learning frameworks have been used to distinguish compensatory versus efficient gait phases, guiding APA decisions on gait reeducation and balance-oriented training [64].

- Personalized APA prescription through ML-based insights

ML models have been used to inform individualized exercise prescription for neuromuscular populations by identifying personal thresholds for fatigue, recommending optimal cadence or stepping frequency, or adjusting resistance levels based on sensor-derived movement quality [63]. In Parkinson’s disease, adaptive cueing systems utilizing ML classify gait abnormalities in real time and adjust external rhythmic cues to improve stability and reduce freezing episodes, applications directly relevant to APA practice [65]. Such adaptive frameworks mirror broader APA principles emphasizing dynamic adjustment of FITT-VP variables in response to day-to-day variability in neuromuscular function [63].

- Real-time monitoring and adaptive safety systems

ML-based anomaly detection, combined with wearables or depth cameras, has facilitated real-time identification of instability, tremor escalation, or sudden gait freezing [8,65]. These systems are particularly promising for home-based or tele-APA models, as they provide continuous monitoring and automated alerts that can enhance safety during unsupervised activity [63]. For individuals with severe or rapidly progressing neuromuscular impairments, ML-enabled monitoring may help regulate intensity, prevent excessive fatigue, and ensure safe transitions between training modalities [63].

- Summary of Key Points

Overall, the literature indicates that ML provides robust support for movement analysis, symptom prediction, functional classification, and individualized exercise adaptation in neuromuscular disorders. By capturing subtle motor abnormalities, compensatory strategies, and day-to-day fluctuations in neuromuscular function, ML enables more precise and responsive APA programming across progressive, fluctuating, and post-lesion conditions. Importantly, ML-based real-time monitoring and adaptive safety systems facilitate the delivery of personalized exercise in home-based and tele-APA contexts, supporting safer progression, optimized workload regulation, and sustained participation in populations characterized by complex and heterogeneous motor profiles. Many applications in neuromuscular disorders remain at the stage of pilot implementation or condition-specific datasets, with limited standardized reporting of long-term deployment in APA contexts.

#### 3.4.6. Adapted Sport

ML applications in adapted sport constitute an emerging yet rapidly growing area, reflecting the technological evolution of performance monitoring and functional classification within Paralympic and disability sport contexts [68,69]. ML can support movement analysis, athlete classification, training optimization, and injury-risk prediction, offering valuable insights for practitioners working across the continuum between APA and adapted sport, particularly in exercise prescription, load management, and functional safety [68,69].

- Movement analysis and performance profiling

Deep-learning and computer-vision techniques have been increasingly used to analyze movement patterns in adapted sports such as wheelchair racing, wheelchair basketball, sitting volleyball, and para-swimming. Markerless pose estimation (e.g., OpenPose, MediaPipe) enables quantification of propulsion mechanics, upper-limb movement symmetry, trunk stability, and sport-specific movement efficiency [6,70]. For wheelchair athletes, ML models trained on IMU and wheel-mounted sensors have been used to detect propulsion technique deviations, energy cost patterns, and movement inefficiencies that may predispose athletes to shoulder strain or performance limitations [68,70]. Methodological advances in ML-based postural and biomechanical assessments provide additional tools for analyzing compensatory strategies and alignment deviations in athletes with motor impairments, with clear implications for training optimization and injury prevention [14].

- Classification and athlete categorization

Functional classification is a core element of Paralympic sport governance. ML has been explored as a tool for improving objectivity and reliability in athlete classification by analyzing biomechanical, physiological, and movement-based features [71]. Neural networks and clustering algorithms have been applied to distinguish levels of functional impairment, classify locomotor patterns, and support the categorization of athletes with cerebral palsy, spinal cord injury, limb deficiency, or neuromuscular disorders [69,71]. These data-driven models complement traditional classification systems by offering quantitative insights that may enhance fairness and transparency.

- Training monitoring and load optimization

Training load management in adapted sport presents unique challenges due to heterogeneity in functional capacity, compensatory strategies, and sport-specific demands. ML models integrating physiological signals, inertial data, and environmental variables have been used to predict training load, fatigue onset, autonomic stress, and performance variation [72,73]. In wheelchair sports, supervised learning models have been applied to detect movement asymmetries and propulsion inefficiencies that increase overuse injury risk [68,70]. For upper-limb–dominant sports, ML-based monitoring of EMG patterns and kinematic metrics has facilitated more precise adjustments to training intensity and technique [14]. Such dynamic adaptations closely resemble APA approaches that rely on individualized progression and real-time regulation of effort, highlighting important conceptual overlaps [69].

- Injury-risk detection and safety monitoring

Wearable-sensor datasets combined with ML algorithms have shown potential in predicting shoulder overuse risk in wheelchair athletes, detecting high-risk postural patterns, and identifying neuromuscular fatigue signatures associated with injury [74,75]. ML-based gait classification has also been applied to assess balance deficits and fall risk in visually impaired and standing Paralympic athletes, informing targeted conditioning exercises within APA programs [72]. These systems are increasingly integrated into remote or hybrid training models, supporting continuous safety monitoring in decentralized training environments [73].

- Bridging adapted sport and adapted physical activity (APA)

A recurring theme is the translational relevance of ML-based insights from adapted sport to broader APA practice. Studies highlight that functional classifications, propulsion mechanics analyses, and movement-quality detection models developed in competitive sport settings can inform adapted exercise interventions for non-athlete populations with similar impairments, improving personalization and safety across settings [70,71]. Moreover, APA frameworks emphasizing individualized load management and functional progression provide a conceptual bridge for transferring ML-enabled performance analytics from elite to community contexts [69,72].

- Summary of Key Points

Overall, ML applications in adapted sport provide robust quantitative tools for movement analysis, functional classification, training-load optimization, and injury-risk detection in athletes with disabilities. Importantly, these approaches extend beyond competitive performance, offering transferable insights for APA practice by supporting individualized load management, safety monitoring, and functional progression. By bridging elite adapted sport technologies with community-based and preventive APA frameworks, ML contributes to a continuum of data-informed exercise interventions that address both performance demands and long-term functional health in people with disabilities. Nonetheless, many adapted-sport ML applications remain validated within sport-specific cohorts, with limited formal evaluation of transferability to broader APA community settings.

To provide an integrated overview of the evidence across conditions, Table 3 summarizes the main ML application targets, key outcome domains, and APA practice implications across primary prevention, disease management, and long-term maintenance pathways, together with indicative study design characteristics and evidence maturity to inform implementation readiness.

### 3.5. Machine Learning-Enhanced Monitoring, Tele-Exercise, and Digital Biomarkers

ML has become integral to modern remote monitoring and tele-exercise systems, providing real-time insights into movement quality, physiological responses, adherence patterns, and functional recovery. These applications are particularly relevant for APA, where continuous supervision is not always feasible and where clinical or functional vulnerability requires data-driven personalization and safety monitoring. Across the reviewed literature, four thematic domains emerged: (i) remote and hybrid exercise delivery systems; (ii) adherence and drop-out prediction; (iii) digital biomarkers of functional status; and (iv) operational applications within community-based APA programs and digitally enabled exercise hubs [11,42].

#### 3.5.1. Remote and Hybrid Exercise Delivery Systems

ML-enabled tele-exercise systems integrate wearable sensors, smartphone-based motion capture, and physiological monitoring to deliver adaptive and safe training sessions in home or community settings. Models trained on accelerometry, gyroscope data, HRV, and respiratory patterns can detect deviations from expected movement quality, early onset of excessive exertion, or signs of instability [34,36,42]. Convolutional and recurrent neural networks have been used to classify repetitions, detect compensatory strategies, estimate training intensity, and provide automated feedback cues to guide safe execution of exercises during unsupervised APA sessions [11]. These systems support scalable, context-aware tele-exercise models, especially for older adults and individuals with chronic disease, reducing reliance on continuous in-person supervision while maintaining safety and personalization [16,42].

#### 3.5.2. Adherence and Drop-Out Prediction

Adherence to APA interventions is a critical determinant of long-term outcomes, yet patterns of engagement often fluctuate due to symptom variability, fatigue, motivation, and environmental constraints. Predictive ML models incorporating activity logs, wearable metrics, and contextual variables (e.g., time-of-day, weather, daily steps) have been used to estimate likelihood of adherence or non-adherence to scheduled exercise sessions [76,77]. Some algorithms can anticipate days with reduced exercise readiness or elevated fatigue burden and suggest adjustments to session content or volume. Recent studies on digital coaching systems highlight how behavioral data streams can provide early indicators of disengagement, enabling APA professionals to address motivational barriers proactively [78].

Drop-out from exercise programs, especially in chronic disease populations, is a major barrier to sustained benefit. ML models using demographic, psychosocial, physiological, and functional indicators have shown strong predictive performance in identifying individuals at higher drop-out risk early in the intervention [79,80]. Features commonly associated with higher risk include irregular activity rhythms, reductions in variability of movement, early declines in HRV, inconsistent training intensity, and psychosocial markers captured through digital diaries. These insights support proactive modifications to APA programs, including motivational strategies, adaptive progression, and individualized follow-up frequencies.

- Ethical and practice considerations

Because adherence and drop-out predictions may influence professional decisions, they should be implemented as supportive “risk flags” (human-in-the-loop) rather than as deterministic labels. Potential risks include stigmatization, biased model errors in underrepresented groups, and self-fulfilling prophecies if individuals are treated as likely to drop out. To mitigate these risks, practitioners should use predictions to increase supportive contact (e.g., check-ins, barrier-solving, motivational interviewing), maintain transparency about data use, avoid punitive responses (e.g., reduced access or intensity solely based on a score), and periodically review model performance and fairness across subgroups [81,82].

#### 3.5.3. Digital Biomarkers of Functional Status and Recovery

Digital biomarkers derived from wearable sensors, smartphones, and video-based movement analysis represent a rapidly expanding field. ML algorithms extract features such as gait variability, tremor dynamics, stride regularity, movement velocity, postural sway, and cardiopulmonary efficiency to quantify functional recovery across diverse chronic conditions [10,33,34]. Longitudinal ML models have predicted improvements in walking performance after structured exercise, recovery trajectories following exacerbations or periods of inactivity, changes in fatigue, dyspnea, autonomic function, and movement efficiency, and individual responsiveness to adapted exercise interventions [11,36,37]. These digital biomarkers complement traditional functional tests by providing continuous, real-world monitoring and by supporting precision adaptation of APA interventions [12,42].

#### 3.5.4. Operational Applications in APA Centers and Community Exercise Hubs

ML-based monitoring systems are increasingly being integrated into APA centers, community exercise hubs, and digitally enabled preventive-health infrastructures. Within these settings, ML technologies support the automated tracking of attendance, training consistency, and exercise intensity, while simultaneously quantifying movement quality during individual or group-based sessions through wearable sensors or computer-vision systems [10,34,83]. In populations with cardiovascular, respiratory, or neuromuscular vulnerabilities, ML-driven safety monitoring enables early detection of abnormal physiological or biomechanical responses, generating alerts that support timely intervention by APA professionals [34].

Beyond individual-level monitoring, ML models applied to real-world activity and behavioral data can be used to predict functional improvement trajectories and adherence patterns, thereby informing personalized progression strategies and allocation of professional supervision [76,79,80]. At an organizational level, aggregated ML outputs allow the development of population-level dashboards that support decision-making, resource allocation, and program optimization within community health networks [81,84]. In practice, dashboards intended for program management may prioritize aggregate indicators (e.g., attendance/adherence trends, distribution of training intensity and workload, alert burden, dropout-risk distribution, and subgroup disparities), whereas dashboards intended for individual supervision emphasize actionable, time-sensitive signals (e.g., safety alerts, abnormal exertion patterns, movement-quality flags, and recovery/fatigue trajectories). Additionally, emerging ML-enabled group-exercise platforms, such as sensor-based exergames, can dynamically adjust workloads in real time based on participants’ sensor feedback, enhancing both safety and inclusiveness in diverse APA groups [19,85].

These operational applications illustrate how ML can be pragmatically embedded within structured APA facilities and community-based exercise programs, supporting scalable, data-informed, and person-centered models of preventive and long-term APA.

- Summary of Key Points

ML-supported monitoring, adherence modeling, and digital biomarkers strengthen the precision, safety, and scalability of APA interventions across home, community, and clinically supervised settings. Across remote and hybrid delivery systems, adherence and drop-out prediction, digital biomarkers, and operational applications in APA hubs, ML enables more responsive exercise regulation, earlier detection of risk signals, and better-informed progression planning. Together, these developments support more accessible and person-centered APA pathways, while preserving professional oversight within tele-exercise and community infrastructures.

For synthesis purposes, Table 4 summarizes the main ML-enabled monitoring, tele-exercise, and digital biomarker applications relevant to APA, including indicative study design characteristics and evidence maturity and practitioner interpretation requirements to inform implementation expectations and safe integration into APA workflows.

### 3.6. Integrating ML in Clinical and Community APA Practice

ML integration into APA practice requires a coordinated strategy across clinical, community, and technological environments. This domain is reported here as a recurrent evidence-based theme describing implementation-oriented use cases across the included literature. Recent literature emphasizes that successful implementation depends on the competencies and decision-making responsibility of APA specialists, kinesiologists, and exercise professionals, infrastructure readiness, and alignment with health-system priorities [1,2,21]. ML-supported systems can enhance decision-making, improve monitoring, and optimize personalization, but they must be embedded within structured APA frameworks to ensure safety and equity [10].

#### 3.6.1. Role of the APA Specialist and Exercise Professionals

APA specialists, kinesiologists, and clinical exercise professionals play a central role in operationalizing ML tools within real-world programs. ML does not replace professional judgment; instead, it acts as a decision-support layer that refines assessment, guides individualized FITT-VP prescriptions, and monitors participant safety [1,21]. Research shows that exercise professionals using ML-derived metrics, such as movement quality indices, digital biomarkers, or predictive fatigue scores, are better equipped to tailor training loads, identify early signs of intolerance, and adjust programs for individuals with chronic conditions or disabilities [6,10]. Key competencies include the ability to interpret wearable-sensor outputs, understand ML-generated classifications or predictions, and translate quantitative signals into actionable exercise decisions [21].

Beyond general digital literacy, essential competency domains include the ability to interpret probabilistic outputs (e.g., risk scores expressed as likelihood rather than certainty), recognize algorithmic limitations such as training-data bias or reduced generalizability to multimorbid APA populations, and override automated recommendations when clinically or functionally indicated. In this framework, ML outputs must be treated as decision-support signals rather than prescriptive commands, with final responsibility remaining with the APA specialist [1,21].

#### 3.6.2. Ambient Intelligence and Sensor-Rich Environments

Ambient intelligence (AmI) refers to environments equipped with interconnected sensors, computer vision systems, and adaptive feedback mechanisms capable of monitoring movement and physiological responses in real time. In APA settings, AmI has been used to detect unsafe compensatory movements, monitor exertional responses during unsupervised tasks, identify instability or fall risk, and provide automated cues for posture correction or pacing [31,32,34]. These intelligent environments extend the reach of exercise professionals, support early risk detection, and enable scalable supervision models applicable in clinics, community centers, and home-based APA programs [10,33]. Recent methodological evidence from ML-based posture and movement analysis supports the feasibility of sensor-rich environments for detecting compensatory strategies and alignment deviations, reinforcing their applicability within APA and community exercise settings [14].

#### 3.6.3. ML-Supported APA Delivery in Community Health and Exercise Hubs

Community-based exercise centers, such as exercise-on-prescription facilities, health-oriented community fitness centers, and kinesiologist-led activity programs, represent ideal settings for ML-supported APA delivery [12,21]. Within these contexts, ML systems can support the routine work of exercise professionals by enabling systematic tracking of attendance and training consistency, objective quantification of movement quality during group sessions through unobtrusive sensors or computer vision, early identification of individuals requiring closer supervision, and dynamic adjustment of group workloads based on real-time functional data [10,33,41]. Moreover, ML facilitates the development of hybrid delivery models that combine in-person supervision with remote monitoring, thereby expanding accessibility while preserving professional oversight [16,42]. These applications align with international trends in preventive health promotion and functional aging and reflect the expanding recognition of APA as an essential component of chronic disease management [22,86].

#### 3.6.4. Clinical–Community Integration

ML can facilitate stronger integration between clinical care and community APA services by enabling the exchange of digital biomarkers, adherence indicators, and functional data [21,42]. This interoperability supports smoother transitions from clinically supervised exercise pathways to long-term APA, including adapted sport where appropriate, while maintaining continuity in exercise objectives, safety thresholds, and progression logic [16]. It also supports shared decision-making between healthcare providers and exercise professionals, stratified referral pathways based on functional risk, and long-term monitoring of high-risk individuals across settings. Such integration addresses a persistent gap in chronic disease management, namely the discontinuity that often occurs when individuals move from hospital-based rehabilitation to community-based, exercise-oriented preventive programs [22,81,84].

#### 3.6.5. Barriers and Facilitators to Implementation

Among the identified barriers, three appear most critical for near-term APA implementation: (1) limited practitioner competence in interpreting ML-derived outputs within FITT-VP decision-making; (2) insufficient external validation of ML tools in heterogeneous APA populations (e.g., multimorbid, older, or community-dwelling participants); and (3) weak interoperability between clinical records and community APA platforms, which impedes continuity of progression and safety thresholds. For example, in community exercise hubs, a lack of integration between wearable dashboards and referral-based APA protocols may prevent timely workload adjustment.

Across the reviewed literature, the implementation of ML within APA practice is influenced by a combination of structural, professional, and technological factors. Several barriers have been consistently identified. Limited digital literacy among exercise professionals may hinder the effective interpretation and contextualization of ML-generated outputs, particularly in the absence of targeted training in data-informed exercise decision-making [87]. In addition, the lack of standardized and clinically validated ML tools specifically designed for APA populations remains a critical challenge, reducing confidence in real-world applicability. Concerns related to data privacy, health-information governance, and regulatory compliance further complicate adoption, especially when sensitive physiological or movement data are involved [88,89]. Financial constraints associated with large-scale deployment of wearable sensors and digital infrastructures also represent a limiting factor, particularly in community-based settings. Finally, insufficient interoperability between clinical systems and community exercise platforms can impede continuity of care and restrict the integration of ML-supported APA services across settings [84,87].

At the same time, the literature highlights several facilitators that may accelerate ML adoption in APA. Structured training programs aimed at improving professionals’ ability to interpret ML outputs and integrate them into exercise prescription and monitoring are increasingly recognized as essential [81]. The growing availability of low-cost, user-friendly wearable technologies has reduced economic and logistical barriers, making ML-supported monitoring more feasible in both clinical and community contexts [12]. The widespread diffusion of open-source computer-vision platforms, such as MediaPipe and OpenPose, has further lowered entry thresholds by enabling scalable movement analysis without reliance on proprietary systems. Moreover, the expansion of tele-exercise models during and after the COVID-19 pandemic has increased familiarity with digital exercise delivery, creating a favorable environment for ML-enhanced approaches [12]. Finally, increasing recognition by health systems and policymakers of APA as a preventive and supportive resource within chronic disease frameworks represents an important institutional driver for sustainable implementation [15,84,86].

- Summary of Key Points

Integrating ML into APA practice requires the coordinated interaction of professional expertise, sensor-based technologies, organizational infrastructures, and clinical–community pathways. Across clinical and community settings, ML functions primarily as a decision-support system that enhances functional assessment, informs individualized FITT-VP prescriptions, and enables continuous safety monitoring, without replacing the judgment of APA specialists and exercise professionals. Sensor-rich and digitally enabled environments extend supervision beyond traditional facilities, supporting hybrid and community-based APA delivery while maintaining clinical oversight. Effective implementation is facilitated by professional training, interoperability between systems, and growing institutional recognition of APA within chronic disease management, whereas barriers related to digital literacy, data governance, and infrastructure remain critical considerations. Overall, ML offers a pragmatic framework for strengthening continuity of care, personalization, and scalability of APA interventions across the lifespan and across levels of functional vulnerability.

### 3.7. Ethical, Legal, and Health-System Considerations

The integration of ML into APA practice raises several ethical, legal, and health-system challenges that must be addressed to ensure safe, equitable, and responsible implementation. These considerations are especially critical when working with individuals with chronic conditions, disabilities, or reduced autonomy, who may be disproportionately affected by algorithmic errors, data misuse, or inequitable access to technological innovations [81,90].

#### 3.7.1. Algorithmic Bias and Fairness

ML models may reproduce or amplify biases present in training datasets. If data used to train movement-analysis or prediction algorithms underrepresent specific populations, such as individuals with severe disabilities, minority groups, or those with multimorbidity, the resulting models may provide inaccurate or discriminatory outputs [91,92]. Examples include underestimation of fall risk in individuals with atypical gait patterns, less accurate fatigue or exertion predictions in underrepresented ethnic groups, and misclassification of movement quality in wheelchair users due to insufficient training data. In APA contexts, bias may also manifest when models trained predominantly on ambulatory gait datasets misclassify wheelchair propulsion patterns as abnormal movement, or when prediction systems calibrated on younger cohorts overestimate exertion tolerance in older or multimorbid participants. Similarly, fatigue-detection models trained on standardized laboratory tasks may underperform in community-based or home-exercise settings characterized by adaptive or compensatory strategies. To mitigate bias, current guidelines recommend transparent reporting of dataset composition, systematic evaluation of model performance across subgroups, and inclusion of diverse populations in model development and validation [87,93].

#### 3.7.2. Equity and Access for Vulnerable Populations

ML-enabled APA systems have the potential to enhance personalization and accessibility, but may also widen inequities when access to devices, connectivity, or digital literacy is limited. Individuals from lower socioeconomic backgrounds, older adults, and people with disabilities often face structural and technological barriers that hinder the adoption of ML-based exercise platforms. Evidence from digital-health research highlights how unequal access to technology, infrastructure, and digital competence can exacerbate disparities in participation and benefit from health innovations [15,94].

Equitable implementation requires affordable or low-resource solutions (e.g., smartphone-based systems), structured training programs to improve digital competence among both participants and professionals, and the design of ML systems that minimize cognitive load and usability barriers. Community partnerships and inclusive program design are also essential to extend access to remote or hybrid APA programs and ensure that individuals with diverse functional, social, and economic profiles can benefit from ML-supported exercise interventions [12].

#### 3.7.3. Privacy, Data Protection, and Handling of Health-Related Data

ML applications in APA routinely process sensitive information, including physiological signals, movement recordings, geolocation patterns, and daily activity metrics. These data raise significant privacy, security, and governance concerns and require compliance with international data-protection frameworks, such as GDPR in Europe and HIPAA in the United States, as well as broader standards for ethical digital-health practice [88,89].

Key requirements include secure storage and encrypted transmission of sensor-derived data, transparent consent procedures that specify data use and retention, and the minimization of personally identifiable information whenever possible. Particular attention is required when using video-based pose estimation systems, which risk capturing identifiable images unless anonymization, on-device processing, or privacy-preserving pipelines are implemented [5]. Ensuring transparency around secondary uses of data, such as model refinement or future research, is essential for ethical deployment of ML within APA.

#### 3.7.4. Professional Responsibility and Liability

As ML tools become integrated into APA practice, the distribution of responsibility between professionals, institutions, and technology providers becomes complex [81,82]. Key questions raised in the literature concern the attribution of responsibility in the event of adverse outcomes following ML-supported exercise prescriptions, particularly when algorithmic recommendations inform intensity or progression decisions. Further issues relate to the extent to which APA professionals are expected to critically validate, contextualize, and potentially override ML-generated outputs within their professional scope of practice.

In practical APA scenarios, liability questions may arise if an ML system recommends an exercise intensity that contributes to an adverse event, particularly when the professional adheres to the suggested parameters. Conversely, uncertainty may also emerge when a professional overrides an algorithmic recommendation and an adverse outcome still occurs. Current ethical and regulatory discussions emphasize that ML outputs should be interpreted as advisory rather than prescriptive, and that professional judgment, including contextual assessment of participant status, comorbidities, and acute symptoms, remains the primary determinant of exercise decisions. Clear documentation of decision rationale and defined escalation pathways are therefore essential safeguards in ML-supported APA practice [82,87].

Finally, there is ongoing debate regarding how ML-driven alerts, risk signals, or anomaly detections should be integrated into existing referrals, escalation, or clinical-decision pathways to ensure timely and appropriate responses without undermining professional accountability. Current ethical frameworks consistently emphasize that ML should function as decision-support technology, not as an autonomous prescriptive agent. Ultimate responsibility for exercise-related decisions remains with qualified professionals, who must understand both the potential and the limitations of ML tools [87,90].

#### 3.7.5. Health-System Implications and Governance

Health systems adopting ML-supported APA services must address a range of governance challenges to ensure responsible and sustainable implementation. These include the certification and validation of ML tools intended for use in preventive and exercise-based care pathways, as well as their technical and semantic interoperability with existing electronic health record systems. In parallel, appropriate reimbursement models for tele-exercise delivery and digital monitoring services need to be defined, together with robust evaluations of cost–effectiveness and long-term sustainability. Finally, governance frameworks should ensure that the deployment of ML-supported APA services remains aligned with population-health priorities and public-interest objectives, rather than being driven predominantly by commercial considerations [81,84]. ML-supported APA programs may contribute to preventive public health strategies, reduction of disability burden, and improved continuity between clinical and community care.

#### 3.7.6. Implications for Community Engagement and Participatory Implementation

ML technologies can expand the reach of APA programs by supporting community health initiatives, inclusion-oriented projects, and collaborative work across health, social, educational, and sport sectors. Ethical and socially responsible implementation requires that communities, including individuals with disabilities or chronic conditions, are actively involved in co-designing and evaluating digital tools to ensure that these systems reflect lived experience and real-world needs [15].

Participatory design in ML-supported APA tools should include structured co-design workshops with end-users (e.g., individuals with disabilities, chronic conditions, or older adults), iterative usability testing in real-world APA environments, and representation of diverse functional profiles in model development datasets. In addition, feedback mechanisms that allow participants and professionals to report misclassifications or inappropriate alerts can support continuous model refinement and contextual adaptation.

Transparency regarding how community-generated data are collected, processed, and used for algorithmic refinement or decision-support is critical. When grounded in participatory and inclusive design frameworks, ML-enhanced APA initiatives can serve as catalysts for equitable health promotion, accessible exercise programs, and socially responsible innovation [12,87].

- Summary of Key Points

Ethical, legal, and health-system considerations are foundational for the responsible integration of ML into APA practice. Key priorities include mitigating algorithmic bias, addressing digital inequalities that limit access to ML-enabled exercise services, and ensuring robust privacy and data-governance safeguards for sensitive movement and physiological data, particularly in video-based systems. The literature also underscores the need for clear professional accountability when ML outputs influence exercise prescription and monitoring, reinforcing that ML should operate as decision-support under qualified oversight. At the system level, sustainable implementation requires validated tools, interoperability, and governance frameworks aligned with population-health goals rather than purely commercial incentives. Finally, participatory and community-engaged design is essential to ensure that ML-supported APA programs remain inclusive, trustworthy, and responsive to real-world needs. Across domains, a consistent gap concerns large-scale external validation, interoperability across systems, and standardized reporting of implementation outcomes.

**Figure 1:**
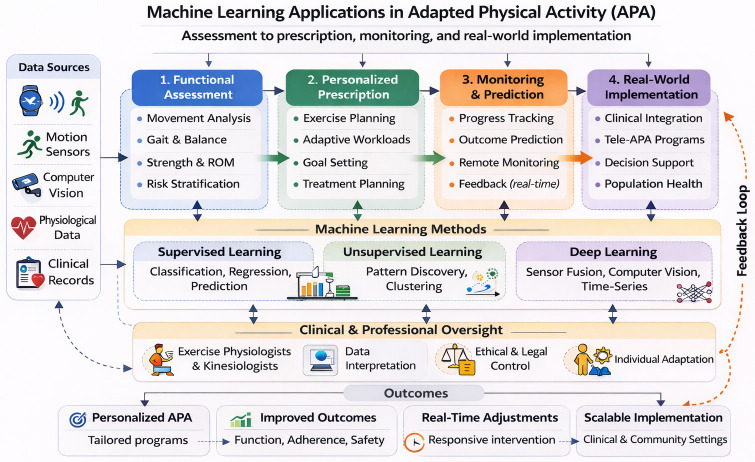
Integrative framework of machine learning applications within adapted physical activity (APA). The figure summarizes how machine learning domains interact across functional assessment, personalized prescription, monitoring and outcome prediction, and real-world implementation within an APA-oriented decision-making pathway. These processes are supported by data-driven methods and professional oversight, with a feedback loop enabling continuous adaptation and outcome optimization. Blue arrows indicate the primary sequential workflow from functional assessment to real-world implementation. Green arrows represent adaptive ML-informed interactions across phases. The orange arrow highlights monitoring-driven translation into real-world application. Bidirectional vertical arrows denote reciprocal exchange between ML methods and professional oversight. The red dashed arrow represents the iterative feedback loop supporting continuous refinement and adaptive APA delivery.

To provide an integrative overview of the findings, Figure 1 summarizes how the identified ML domains interact within an APA-oriented decision-making pathway. The figure illustrates the relationships among functional assessment, personalized prescription, monitoring and outcome prediction, and real-world implementation, supported by data-driven systems and professional expertise.

## 4. Discussion

The findings of this narrative review indicate that ML can meaningfully enhance APA by strengthening functional assessment, supporting individualized exercise prescription, improving safety monitoring, and enabling scalable delivery across clinical and community pathways. Across chronic conditions, disability contexts, and adapted sport environments, ML-derived outputs, such as digital biomarkers, predictive indicators of fatigue or tolerance, movement-quality metrics, and adaptive feedback, support a shift toward more responsive, data-informed exercise interventions grounded in real-world functional data.

A central and cross-cutting theme emerging from the reviewed evidence is that ML does not replace professional expertise. Rather, it extends the analytical capacity of APA specialists, kinesiologists, and exercise professionals by providing continuous and context-aware information that is difficult to obtain through conventional assessment alone. When embedded within structured APA frameworks and safety procedures, ML can reinforce professional judgment in populations characterized by heterogeneity of functional capacity, symptom fluctuation, and complex clinical profiles [81,87]. Importantly, the relevance of ML in this field depends on its implementation as decision support, with clear professional accountability and validated operating thresholds, especially when exercise decisions are influenced by automated classification or risk signals [82]. However, determining the operational boundary between reliance on ML-generated signals and professional experiential judgment remains an open empirical and ethical question. Future research should examine how professionals calibrate trust in probabilistic outputs under varying levels of uncertainty, and how hybrid decision models can optimize safety without diminishing clinical reasoning.

Beyond the current applications summarized in the results, the literature points to several trajectories that may shape future ML integration in APA. These directions should be interpreted as extensions of existing practice, rather than disruptive replacements, and their translation into routine APA services will depend on validation, feasibility, governance, and professional training. In addition to technical validation, key barriers include interoperability across digital health platforms, regulatory uncertainty surrounding ML-supported decision-making, limited reimbursement frameworks, and variability in digital literacy among APA professionals and end users.

### 4.1. Digital Twins and Individualized Exercise Modeling

The following domains represent emerging extensions of currently validated monitoring and prediction systems, with increasing levels of technical and regulatory uncertainty. Digital twins, i.e., computational representations of individual physiological and biomechanical systems, represent a promising conceptual direction for personalized exercise planning. By integrating wearable-derived signals, functional metrics, and ML-based prediction models, digital twins may support simulation of individual responses to different training loads, intensities, or movement strategies. In APA contexts, this could help anticipate fatigue, tolerance, and recovery patterns before implementing interventions, with potential benefits for safety and efficiency in populations with fluctuating symptoms or reduced physiological reserve. While still emerging, this trajectory is consistent with the broader expansion of unobtrusive, continuous monitoring and real-time physiological modeling reported across current ML applications in exercise and health systems [63,81].

### 4.2. ML-Enhanced VR and AR Exercise Environments

Virtual and augmented reality environments enriched with ML-driven movement analysis may further improve engagement, motor learning, and execution quality during adapted exercise. The convergence of computer vision, wearable sensing, and adaptive feedback suggests that ML-enabled VR/AR platforms could become practical tools within supervised and home-based APA programs, particularly for balance, gait retraining, and cognitive–motor tasks. Importantly, these technologies should be viewed as complements to professional supervision rather than substitutes, with ML used to support real-time feedback, safety monitoring, and individualized progression [18,19].

### 4.3. Ambient Intelligence and Sensor-Rich Environments

Ambient intelligence systems, characterized by embedded sensors and adaptive ML algorithms, may facilitate continuous monitoring of movement, exertion, and safety signals in real-world settings. In applied APA environments, sensor-rich infrastructures can support automated detection of instability, compensatory strategies, or excessive load, and can generate alerts that help professionals intervene early when risks emerge. This direction aligns with the growing feasibility of unobtrusive sensing, multimodal integration, and real-world functional monitoring highlighted in the Results, and it may enable broader scaling of community-based APA services while preserving professional oversight [33,88].

### 4.4. Functional Forecasting and Predictive Recovery Models

ML-based forecasting models may support prediction of recovery trajectories, exercise tolerance, exacerbation risk, and functional decline across chronic conditions. By enabling early identification of non-responders and dynamic adaptation of FITT-VP variables based on predicted readiness, these approaches may improve both safety and efficiency, especially when exercise participation occurs in decentralized settings with limited face-to-face supervision. In APA services, forecasting can support practical decisions such as when to reduce intensity, modify modality, increase supervision, or adjust progression rates, particularly in populations characterized by symptom variability or unstable physiological responses [36,37].

### 4.5. Next-Generation Wearable Biosensors and Multimodal Monitoring

The evolution of wearable technologies toward multimodal, unobtrusive, and lower-cost systems is likely to further expand ML applications in APA. Integration of cardiac, respiratory, neuromuscular, metabolic, and biomechanical signals may provide more complete profiles of movement quality and physiological stress, enabling derivation of functional biomarkers and more precise safety monitoring in remote and hybrid exercise models. As wearable platforms mature, the clinical relevance of these signals will depend on validation, interpretability, and the establishment of governance and accountability frameworks to ensure safe and equitable deployment [56,87]. At present, these approaches remain promising but require broader external validation, cost–effectiveness evaluation, and governance clarification before large-scale adoption.

### 4.6. Overall Implications for APA Practice and Research

The integration of ML into APA represents a movement toward more individualized, data-informed, and scalable exercise pathways [95]. Future work should prioritize multi-center validation, transparency and interpretability of models, and implementation research that explicitly integrates professional workflows, data governance, and equity considerations. Priority research directions should also include prospective comparative trials within structured APA programs, standardized reporting of deployment metrics, cost-effectiveness analyses, and long-term evaluation of safety and adherence outcomes. When aligned with professional expertise and community infrastructures, ML may support improvements in the precision and accessibility of exercise-based interventions across chronic conditions and disability contexts.

From a practical perspective, wearable-supported monitoring systems, supervised classification of functional impairments, and predictive modeling of physiological responses based on validated sensor platforms represent applications closest to routine APA integration. In contrast, fully autonomous closed-loop prescription systems, digital twin modeling, and large-scale AI-driven risk stratification remain emerging and require further external validation before widespread implementation.

Taken together, the reviewed evidence suggests that several ML applications are approaching a level of technical readiness suitable for pilot implementation in APA services, while broader adoption will depend on professional training, infrastructure availability, and alignment with existing care pathways. Conceptually, the proposed framework integrates four interconnected components: (i) data inputs derived from wearable, sensor-based, and contextual sources; (ii) ML functions supporting classification, prediction, and pattern recognition; (iii) APA decision-making, including assessment, prescription, and monitoring guided by professional judgment; and (iv) contextual and governance layers, encompassing implementation settings, ethical safeguards, and accountability mechanisms.

#### 4.6.1. Research Gaps

Among the identified gaps, three priorities are most critical for safe implementation: (1) large-scale external validation across heterogeneous APA populations; (2) integration of ML outputs into structured professional workflows with defined accountability thresholds; and (3) prospective trials evaluating safety, adherence, and cost–effectiveness in real-world APA services. Addressing these priorities would have the greatest immediate impact on translational feasibility and clinical trust.

#### 4.6.2. What This Review Adds

From an APA and kinesiology perspective, this review extends existing literature by providing an integrated, APA-specific synthesis of ML applications that moves beyond technical performance metrics. First, it consolidates evidence on ML-enhanced functional assessment, highlighting scalable tools for movement analysis and risk stratification across clinical and community settings. Second, it clarifies how ML can support personalized exercise prescription and progression through prediction of tolerance, fatigue, and functional responsiveness. Third, it frames ML-enabled monitoring, tele-exercise, and digital biomarkers as practical mechanisms for adaptive regulation and safety management in real-world APA pathways, emphasizing professional oversight and implementation feasibility.

### 4.7. Methodological Considerations and Limitations

As with all narrative reviews, some methodological limitations should be considered when interpreting the findings. Although the search strategy was structured and multi-database, it does not ensure exhaustive coverage of all relevant publications. The inclusion of computer science and engineering literature increased conceptual breadth but also introduced heterogeneity in study designs and outcome reporting. Furthermore, the thematic synthesis adopted in this review involved interpretative judgment in defining conceptual domains and relevance. In line with the nature of narrative reviews, no formal risk-of-bias assessment or quantitative appraisal was conducted, limiting direct comparison of methodological quality across studies [23].

The 2018 start date may have excluded foundational ML applications in biomechanics or digital health that informed later APA developments. Evidence quality varies substantially across conditions and applications, ranging from exploratory feasibility studies to externally validated predictive models. Finally, publication bias toward positive or technically successful ML findings may inflate perceived readiness for implementation, warranting cautious interpretation.

In addition, citation bias cannot be fully excluded, as studies reporting positive or innovative ML applications may be more likely to be published and cited. To mitigate potential over-representation of engineering-driven contributions, evidence was interpreted through an APA-oriented lens, prioritizing functional relevance, applicability to adapted exercise settings, and alignment with professional decision-making rather than algorithmic performance alone.

## 5. Conclusions

ML is emerging as a transformative extension of the toolkit available to APA specialists and kinesiologists, augmenting, rather than replacing, professional judgment in functional assessment, exercise prescription, and monitoring across chronic conditions. By supporting more sensitive detection of movement impairments, improving estimation of exercise tolerance, enabling remote supervision, and informing data-driven adjustments of FITT-VP variables, ML contributes to safer, more adaptive, and more scalable exercise interventions.

From a practitioner perspective, current evidence supports the near-term integration of wearable-based monitoring systems, validated gait and movement classification tools, and predictive models of physiological tolerance within structured APA programs. Exercise professionals should prioritize competency in interpreting probabilistic ML outputs, documenting decision rationale, and integrating ML-derived signals into established FITT-VP frameworks while retaining clinical oversight.

From a research perspective, priorities include large-scale external validation across heterogeneous APA populations, prospective safety and effectiveness trials embedded in real-world services, and improved reporting of interpretability, workflow integration, and cost–effectiveness outcomes.

From a policy and health-system perspective, implementation efforts should focus on interoperability standards, reimbursement models for ML-supported monitoring, certification pathways for digital tools, and governance frameworks that ensure equity, accountability, and robust data protection.

Importantly, wearable-supported monitoring, supervised functional classification, and validated predictive models are closest to implementation readiness. In contrast, fully autonomous closed-loop prescription systems, digital twin modeling, and large-scale AI-driven risk stratification remain emerging applications that require further external validation, regulatory clarification, and implementation research before widespread adoption.

Over the next 3–5 years, responsible ML-enhanced APA practice should be characterized by hybrid decision-making models in which professionals retain ultimate accountability; validated and interoperable monitoring systems embedded within clinical–community pathways; transparent governance and equity safeguards; and continuous feedback loops that refine models through real-world data. In this model, ML functions as an accountable decision-support infrastructure that strengthens professional expertise, improves safety, and expands equitable access to APA across diverse populations.

## Figures and Tables

**Table 1:** Conceptual overview of major machine learning families with APA-oriented examples.

ML Family	Typical Input Data in APA	Core Analytical Logic	Representative APA Use Cases	Typical Outputs for Practice
**Supervised learning**	IMU-derived gait features; HR/HRV; labeled video tasks; clinical variables	Learns from labeled datasets to classify or predict predefined outcomes	Fall-risk classification; fatigue prediction; exercise tolerance estimation; gait impairment detection	Risk categories; predicted values; decision-support indicators
**Unsupervised learning**	Wearable time series; multidimensional kinematic features; activity logs	Identifies latent patterns or subgroups without predefined labels	Mobility phenotype identification; clustering of functional profiles; detection of atypical motor patterns	Clusters/phenotypes; movement profiles; stratification cues
**Deep learning**	Raw RGB/depth video; raw IMU signals; multimodal physiological streams	Automatically learns hierarchical representations from high-dimensional data	Markerless pose estimation; movement segmentation; automated movement-quality scoring	Joint coordinates; task segmentation; movement-quality indices

Notes. APA = adapted physical activity; IMU = inertial measurement unit; HR = heart rate; HRV = heart rate variability; RGB = red–green–blue video.

**Table 2:** Main domains of ML applications in APA for functional assessment: data sources, analytical approaches, functional outcomes, practice relevance, and indicative study design characteristics, validation metrics, context, and sample-size ranges to support interpretation of implementation readiness.

ML Application Domain	Primary Data Sources	Main ML Approaches	Functional Outcomes Assessed	Relevance to APA Practice	Study Design Characteristics and Evidence Maturity	Key References
**Automated functional movement assessment**	IMUs, accelerometers, gyroscopes, RGB video	Supervised learning, deep learning	Gait symmetry, movement quality, postural stability	Objective functional screening and monitoring across clinical and community settings	Exploratory–internal validation; small pilot or single-cohort studies (n ≈ 20–80); typical accuracy 75–90%; predominantly laboratory or controlled clinical settings	Zago et al., 2021 [2]; Roggio et al., 2024 [14]
**Markerless motion analysis**	RGB video, depth cameras	Pose estimation, CNN-based models	Joint kinematics, task execution quality	Community- and home-based movement evaluation	Internal validation; limited external validation; mostly laboratory datasets (n ≈ 15–60); joint-angle errors typically ~3–8°; rare community-based validation	Wade et al., 2022 [5]; Roggio et al., 2024 [6]
**Automated gait analysis**	Wearable sensors (IMUs), video	Classification, deep learning	Gait variability, spatiotemporal parameters, symmetry	Identification of subtle functional impairments and progression monitoring	Internal–external validation (condition-dependent); heterogeneous sample sizes (n ≈ 30–150); reported classification accuracy often 80–95%; mainly laboratory or supervised clinical datasets; limited real-world APA deployment	Bailo et al., 2024 [7]; Sumner et al., 2023 [21]; Roggio et al., 2024 [14]
**Balance and fall-risk assessment**	IMUs, accelerometry, video	Neural networks, ensemble models	Postural sway, stability indices	Risk stratification in vulnerable populations	Internal validation; limited prospective validation; sample sizes typically n ≈ 25–120; accuracy/sensitivity often 75–92%; mostly laboratory or clinical environments	Chen et al., 2022 [31]; Lin et al., 2022 [32]
**Multimodal functional monitoring**	Motion + physiological sensors (HR, HRV, EMG, respiration)	Sensor fusion, time-series DL	Movement quality, exertional response, functional tolerance	Continuous functional monitoring during APA programs	Exploratory–internal validation; feasibility or proof-of-concept studies (n ≈ 15–70); performance metrics variable; predominantly laboratory or short-term supervised implementations	Raihan et al., 2025 [11]; Souaifi et al., 2025 [10]
**Functional stratification and risk profiling**	Long-term wearable data	Unsupervised learning, supervised classification	Mobility phenotypes, functional heterogeneity	Individualized program planning and risk-informed progression	Exploratory–internal validation; retrospective wearable datasets (n ≈ 40–200); clustering validity reported via internal indices; mostly laboratory-derived or secondary datasets	Hackett et al., 2024 [33]; Chen et al., 2022 [31]; Zmudzki et al., 2025 [4]

Notes. Study design characteristics, validation context (e.g., laboratory, clinical, community-based), evidence maturity, typical validation metrics, and approximate sample-size ranges are reported as qualitative, indicative descriptors derived from representative studies within each domain. These descriptors are intended to support interpretation of implementation readiness and translational applicability, and do not constitute a formal risk-of-bias assessment or meta-analytic synthesis. Abbreviations: APA = adapted physical activity; IMU = inertial measurement unit; HR = heart rate; HRV = heart rate variability; EMG = electromyography; DL = deep learning; CNN = convolutional neural network; RGB = red–green–blue (standard color video).

**Table 3:** ML applications across chronic conditions and adapted sport: targets, outcome domains, APA practice implications, and indicative study design characteristics and evidence maturity to inform implementation readiness.

Condition/Context	Main ML Application Targets	Key Outcomes/Markers	APA Practice Implications	Study Design Characteristics and Evidence Maturity	Key References
**Oncology**	Fatigue prediction; functional decline; movement signatures	Cancer-related fatigue, tolerance, deconditioning	Progression planning; workload modulation; adherence support	Internal validation; limited real-world deployment; primarily small-scale or single-center cohorts	Beenhakker et al., 2025 [39]; Wang et al., 2024 [40]; Chen et al., 2024 [44]
**Metabolic disorders/Obesity**	Cardiorespiratory fitness estimation; glycemic prediction; gait deviations	VO_2_max estimation; postprandial glucose; exertion variability	Individualized intensity zones; low-impact progression; safety thresholds	Exploratory–internal validation; often retrospective or wearable-based feasibility studies	Frade et al., 2023 [41]; Hotta et al., 2024 [48]; Namazi et al., 2025 [35]
**Cardiovascular diseases**	Exercise intolerance prediction; BP estimation; VT estimation; HF decompensation alerts	HR/BP dynamics; VT; decompensation risk	Risk-managed prescription; remote monitoring; safer tele-APA	Internal–external validation (use-case dependent); mixed prospective and retrospective designs with heterogeneous datasets	Kato et al., 2025 [53]; Min et al., 2025 [56]; Contreras-Briceño et al., 2024 [57]; Saha et al., 2025 [58]
**Respiratory diseases (COPD/asthma/ILD)**	Ventilatory response prediction; dyspnea monitoring; exacerbation risk	SpO_2_ trends; dyspnea patterns; ventilatory instability	Symptom-adapted FITT-VP; remote supervision; individualized safety limits	Exploratory–internal validation; limited large-scale prospective APA trials	Shah et al., 2023 [59]; Liao et al., 2021 [60]; Contreras-Briceño et al., 2024 [57]
**Neuromuscular disorders**	Gait abnormality detection; FoG prediction; progression monitoring	Freezing of gait, tremor dynamics, mobility decline	Adaptive cueing; safety alerts; individualized progression	Internal validation; limited real-world deployment; frequently condition-specific pilot implementations	Bouchouras et al., 2025 [65]; Zhao et al., 2023 [67]; Gu et al., 2026 [63]
**Adapted sport/disability sport**	Sensor-based load monitoring; classification support; injury-risk prediction	Propulsion metrics; asymmetries; overuse risk	Load management; functional safety; transfer to community APA	Exploratory–internal validation; validated mainly within sport-specific cohorts rather than community APA settings	Rum et al., 2021 [68]; Wileman et al., 2025 [71]; Weizman et al., 2024 [70]; Leckey et al., 2025 [74]

Notes. Study design characteristics and evidence maturity are summarized qualitatively (e.g., pilot, retrospective, prospective, internal validation, external validation, real-world implementation) and reflect typical evidence patterns within each condition/domain, rather than a formal risk-of-bias or systematic appraisal of individual studies. Across conditions, most evidence primarily reflects disease-management and rehabilitation-adjacent APA contexts, with comparatively fewer studies addressing primary prevention or long-term maintenance in community programs. Underrepresented groups commonly include older adults with multimorbidity/frailty, socioeconomically disadvantaged populations, and individuals with severe disability or advanced disease stages, limiting generalizability to some APA service models. Abbreviations: APA = adapted physical activity; COPD = chronic obstructive pulmonary disease; ILD = interstitial lung disease; VO_2_max = maximal oxygen uptake; VT = ventilatory threshold; SpO_2_ = peripheral oxygen saturation; BP = blood pressure; HF = heart failure; HR = heart rate; FoG = freezing of gait.

**Table 4:** ML-enabled monitoring, tele-exercise systems, and digital biomarkers in APA: components, monitored domains, implementation functions, and indicative study design characteristics and evidence maturity, and practitioner interpretation requirements.

ML-Enabled Application Area	Core Data Sources	Main ML Functions	Key Monitored Domains	APA Implementation Relevance	Study Design Characteristics and Evidence Maturity	Practitioner Interpretation Requirements	Key References
**Remote and hybrid tele-exercise delivery**	Wearable sensors (IMUs, HR, HRV), smartphone video	Activity recognition; movement-quality classification; intensity estimation	Exercise execution, movement quality, exertional response	Safe delivery of home-based and hybrid APA with reduced need for continuous supervision	Internal validation; limited pragmatic evidence; primarily feasibility or pilot implementation studies	Interpret basic wearable/video metrics; apply safety thresholds; know escalation pathways	Klein et al., 2025 [42]; Raihan et al., 2025 [11]
**Automated feedback and real-time supervision**	IMUs, RGB video, physiological signals	CNNs, RNNs, anomaly detection	Compensatory movements, instability, excessive exertion	Real-time correction and safety monitoring during unsupervised APA sessions	Exploratory–internal validation; technology-driven prototypes with limited external validation	Understand model-generated feedback and false alarms; decide when to intervene/modify exercise	Raihan et al., 2025 [11]; Wei & Wu, 2023 [34]
**Adherence and drop-out prediction**	Activity logs, wearable metrics, contextual data	Supervised prediction models	Session adherence, engagement patterns, fatigue-related non-participation	Early identification of disengagement risk and personalized motivational strategies	Internal validation; limited prospective testing; mostly retrospective or short-term prospective datasets	Use risk scores ethically (supportive outreach, not punitive); interpret uncertainty and subgroup bias	Choe et al., 2025 [76]; Ekpezu et al., 2023 [77]; Hegde et al., 2024 [78]
**Digital biomarkers of functional status**	Wearables, smartphones, video-based motion capture	Feature extraction; longitudinal ML models	Gait variability, postural control, functional recovery, fatigue	Continuous functional monitoring and precision adjustment of APA programs	Exploratory–internal validation; few longitudinal multi-site validations	Translate biomarker trends into FITT-VP adjustments; communicate meaning to participants	Hackett et al., 2024 [33]; Wei & Wu, 2023 [34]; Souaifi et al., 2025 [10]
**Risk detection and safety alerts**	Motion + physiological sensors	Anomaly detection; risk classification	Abnormal exertion, instability, physiological stress	Safety management in vulnerable populations during community and home APA	Exploratory–internal validation; few longitudinal multi-site validations	Respond to alerts with clinical judgement; confirm signals; document actions and follow-up	Wei & Wu, 2023 [34]; Raihan et al., 2025 [11]
**Operational deployment in APA centers and community hubs**	Wearables; computer vision; attendance data	Aggregated ML analytics	Attendance, training consistency, workload distribution	Program optimization, scalable supervision, and resource allocation	Emerging real-world use; limited evaluation; early-stage implementation studies without controlled comparative designs	Interpret dashboards; basic data governance literacy; integrate insights into staffing/protocol decisions	Espinosa et al., 2025 [83]; Bhuyan et al., 2025 [84]; Topol, 2019 [81]
**ML-enabled group exercise and exergames**	Motion sensors; physiological feedback	Adaptive workload algorithms	Group intensity balance, inclusiveness, safety	Dynamic adaptation of group-based APA sessions	Exploratory; proof-of-concept or small cohort evaluations	Oversee algorithm-driven intensity changes; ensure inclusiveness and safety rules are met	Au et al., 2025 [85]; Greco et al., 2025 [19]

Notes. Study design characteristics and evidence maturity are summarized qualitatively (e.g., feasibility study, pilot implementation, retrospective dataset, prospective validation, external validation, early real-world deployment) to contextualize implementation readiness. These descriptors are indicative and do not replace formal risk-of-bias assessment or systematic methodological grading of individual studies. Practitioner interpretation requirements are provided as concise, practice-oriented descriptors of the minimum competencies needed to interpret ML outputs and act safely within APA programs. Abbreviations: APA = adapted physical activity; IMU = inertial measurement unit; HR = heart rate; HRV = heart rate variability; CNN = convolutional neural network; RNN = recurrent neural network; RGB = red–green–blue video.

## Data Availability

No new data were created or analyzed in this study.

## Supplementary Materials

The following supporting information can be downloaded at: https://www.mdpi.com/article/10.3390/jfmk11010106/s1, Table S1: Complete database-specific search strategies used in the literature search.
