# Supplementary material for: Machine Learning in Adapted Physical Activity: Clinical Applications, Monitoring, and Implementation Pathways for Personalized Exercise in Chronic Conditions: A Narrative Review
Source: J Funct Morphol Kinesiol. 2026 Mar 4;11(1):106. doi: 10.3390/jfmk11010106 (PMC13028406; doi:10.3390/jfmk11010106)
Supplement: Supplementary file 1 [file jfmk-11-00106-s001.zip › jfmk-4146766-supplementary.pdf]

Supplementary Table S1

Complete database-specific search strategies used in the literature search

PubMed/MEDLINE

("machine learning"[Title/Abstract] OR "artificial intelligence"[Title/Abstract] OR "deep learning"[Title/Abstract] OR "neural network*"[Title/Abstract])
AND
("adapted physical activity"[Title/Abstract] OR "exercise prescription"[Title/Abstract] OR "exercise therapy"[Title/Abstract] OR "functional assessment"[Title/Abstract] OR "rehabilitation exercise"[Title/Abstract])
AND
("gait"[Title/Abstract] OR "balance"[Title/Abstract] OR "posture"[Title/Abstract] OR "movement analysis"[Title/Abstract] OR "wearable sensor*"[Title/Abstract] OR "pose estimation"[Title/Abstract])
Filters: 2018–2026; Humans; English

Scopus

TITLE-ABS-KEY("machine learning" OR "artificial intelligence" OR "deep learning" OR "neural network*")
AND
TITLE-ABS-KEY("adapted physical activity" OR "exercise prescription" OR "functional assessment" OR "exercise therapy")
AND
TITLE-ABS-KEY("gait" OR "balance" OR "posture" OR "movement analysis" OR "wearable sensor*" OR "digital biomarker*")
AND PUBYEAR > 2017

Web of Science

TS=("machine learning" OR "artificial intelligence" OR "deep learning" OR "neural network*")
AND
TS=("adapted physical activity" OR "exercise prescription" OR "functional assessment")
AND
TS=("gait" OR "balance" OR "posture" OR "wearable sensor*" OR "pose estimation")
Timespan: 2018–2026; Document types: Article, Review

IEEE Xplore

("machine learning" OR "deep learning" OR "artificial intelligence")
AND
("gait" OR "balance" OR "movement analysis" OR "wearable sensors")
AND
("exercise" OR "rehabilitation" OR "adapted physical activity")
Publication Years: 2018–2026

ACM Digital Library

("machine learning" OR "deep learning")
AND
("human movement" OR "gait" OR "balance")

AND
("exercise" OR "rehabilitation")
Publication Date: 2018–2026

Google Scholar
Used combinations of:
"machine learning" + "adapted physical activity"
"machine learning" + "exercise prescription"
"deep learning" + "gait" + "rehabilitation"
Screened first 200 results sorted by relevance.
